# Femtosecond imaging of spatial deformation of surface plasmon polariton wave packet during resonant interaction with nanocavity

**DOI:** 10.1515/nanoph-2021-0740

**Published:** 2022-02-25

**Authors:** Naoki Ichiji, Yuka Otake, Atsushi Kubo

**Affiliations:** Graduate School of Pure and Applied Sciences, University of Tsukuba, 1-1-1 Tennodai, Tsukuba-shi, Ibaraki, 305-8571, Japan; Graduate School of Pure and Applied Sciences, University of Tsukuba, 1-1-1 Tennodai, Tsukuba-shi, Ibaraki, 305-8571, Japan; Faculty of Pure and Applied Sciences, University of Tsukuba, 1-1-1 Tenno-dai, Tsukuba-shi, Ibaraki, 305-8571, Japan

**Keywords:** femtosecond, metamaterial, nanocavity, surface plasmon, time-resolved microscopy, wave packet

## Abstract

The spatiotemporal dynamics of a surface plasmon polariton (SPP) wave packet (WP) that interacts with a plasmonic nanocavity on a metal surface are investigated via femtosecond time-resolved two-photon fluorescence microscopy and numerical calculations. The nanocavity, which consists of a metal–insulator–metal (MIM) laminar structure (longitudinal length: ∼100 nm), behaves as a subwavelength meta-atom possessing discretized eigenenergies. When a chirp-induced femto-second SPP WP is incident on the nanocavity, only the spectral component matching a particular eigenenergy is transmitted to continue propagation on the metal surface. This spectral clipping induces a spatial peak shift in the WP. The shift can be controlled by tuning the eigenenergy or chirp.

## 1 Introduction

In optical physics, the control of the spatiotemporal dynamics of light pulses has been a fascinating topic of study. The tunability of material dispersion and/or optical resonance has facilitated control over group velocities of light in a variety of natural materials and artificial nanostructures, including gaseous atoms [1, 2], ultracold atoms [3], optical fibers [4], ring resonators [5, 6], photonic crystals [7, 8], plasmonic Bragg gratings [9], and metamaterials [10], [11], [12], [13]. Metamaterials consist of arrayed subwavelength-scaled optical resonators, each of which provides specific shifts in the phase, amplitude, and polarization between the incident and scattered components of the light field [14]. Accordingly, light has been tailored to realize various applications such as anomalous refraction and reflection [15], vector beam formation [16], spectral filtering [17], light acceleration [18, 19], and ultrafast optical pulse shaping [20].

An established method for achieving control of the group velocity of light is the use of spectral windows in which materials exhibit abnormal dispersion responses to frequency [21], [22], [23]. In the framework of classical electrodynamics, the realization of group velocities that deviate significantly from the speed of light in vacuum, such as subluminal, superluminal, and negative group velocities in natural materials, has been interpreted as a result of the steep change in the refractive index near the absorption lines of atoms [1], [2], [3]. Slow light in photonic crystals has been caused by a large bending of the photonic band near the Brillouin zone edge [7], [8], [9]. Doubly negative phase and group velocities in metamaterials have been realized by using regions with negative values of both permittivity and permeability near the electric and magnetic resonance frequencies of the artificial resonator structure [10]. The width of the spectral window, in which these peculiar optical pulse propagations occur, is usually not very wide. Spectral widths of light pulses should be narrow enough to fit within the limited frequency range. In these cases, the instantaneous frequency of the light pulse can be assumed to be constant over the pulse duration. The envelope shape of the pulse remains similar before and after transmission through the natural materials or the artificial structures, although the amplitude would be attenuated because of the absorption by materials.

If the optical pulse has a large spectral width that exceeds the spectral window described above, and multiple resonance lines enter the spectral width, the amplitude and phase modulation of each of the frequency component of the pulse will be significantly different for each frequency. In this case, the pulse waveform of the incident light is no longer maintained, because both the envelope shape and the instantaneous carrier frequency are modulated significantly in time. This situation is rather similar to the pulse shaping of femtosecond laser pulses [20, 24].

Recently, several methods using spatio-temporal couplings (STCs) [25, 26] or space-time correlations [27, 28] of light pulses have been reported, which allow spatio-temporal control of light pulses based on a different principle rather than relying on the spectral window of materials. In these methods, the direction of wavevector is controlled for each frequency component of the light pulse. The spectral widths of light pulses are no needed to be kept narrow. By shaping the spatial distribution of the pulse in both the longitudinal and the transverse directions, the temporal evolution of the peak intensity of the optical pulse can be highly controlled. Moreover, by exploiting the degree of freedom in the temporal variation of the carrier frequency of optical pulses, advanced spatio-temporal control of light has been achieved, including a wide-range control of the light group velocity [29], [30], [31], temporal modulation of the light velocity [32, 33], bending and steering of the light direction [34], and pulses propagations in a dispersive medium without spreading [35].

We recently reported a finite-difference time-domain (FDTD) simulation study of the deformation of the temporal waveform of a surface plasmon polariton (SPP) wave packet (WP) with a femtosecond time duration and a wide spectral width transmitted through a metal–insulator–metal nanocavity (MIM-NC) [36]. The MIM-NC is a typical meta-atom used in the visible to infrared light regions [37], [38], [39], [40], [41]. The levels of eigenenergies were controlled by tuning the structural length of the MIM-NC. When an up-chirped, broadband SPP WP was incident, the spectral components overlapping the cavity eigenmodes were transmitted otherwise they were reflected. The transmitted SPP WP still maintained a femtosecond time duration due to a wide linewidth of the eigenmodes, however, the coordinate of the intensity peak showed a significant shift in time compared to a reference WP which propagated on a flat surface without the MIM-NC. The shift of the intensity peak was controlled either forward or backward by tuning the eigenenergy of the MIM-NC. If the position of the wave packet is defined as the coordinate of the maximum intensity of the envelope, then this peak shift produces an apparent velocity anomaly as the wave packet passes through the nanocavity. The temporal behavior of SPP WPs were tuned by controlling the direction of the wavevector of each frequency component using cavity resonances, which could be conceptually compared to the spatio-temporal control of light pulses using STCs. In metamaterials, the optical responses of the meta-atoms are accumulated over the spectral bandwidth and sectional area of the incident light beam to determine the complete spatiotemporal behavior of the light pulses [42]. Therefore, to realize emerging applications based on the newly developed controllability of light, it is indispensable to understand the dynamic responses of individual meta-atoms and their roles in the modulation of light pulses in terms of intensity, spatial shape, and temporal shape [43, 44].

In this study, the spatiotemporal dynamics of SPP WPs interacting with a subwavelength-scale MIM-NC were investigated by femtosecond time-resolved two-photon fluorescence microscopy (TR-2PFM). 10 fs duration and 810 nm center wavelength light pulses irradiate an Au film containing a coupler and an MIM-NC to initiate SPP WP propagation [45]. The SPP WPs were optically imaged by up-converting a small portion of the energy of the SPP field to fluorescent emission with the wavelength of about 470 nm through two-photon fluorescence process in a ∼50 nm thick dye-doped PMMA layer coated on the surface [46], [47], [48], [49]. In combination with a pump-probe technique, spatio-temporal evolution of SPP WP was dynamically visualized with 10 fs time and 0.5 μm spatial resolutions. The TR-2PFM provided time-resolved movies of SPP WPs that were similar character to those produced by time-resolved photoemission electron microscopy (TR-PEEM), which has been a representative method for revealing SPP dynamics on femtosecond timescales [50], [51], [52], [53], [54], [55], [56], [57]. Although the spatial resolution of the TR-2PFM is limited by the resolution of the objective lens of the optical microscope and thus it is inferior to that of a PEEM, the TR-2PFM has several useful characteristics: The measurements can be performed in air, and the sample material can be a variety of materials including insulators. In addition, TR-2PFM can be easily combined with a variety of light sources such as infrared lasers and vector beams.

The TR-2PFM images revealed the motion and deformation of the SPP WPs, which evolved as a function of the pump–probe delay time. The coordinates of the intensity peak of a WP showed a distinct spatial shift relative to the coordinates of a reference peak when the WP was transmitted through an MIM-NC. The spatial shifts were controlled to within several micrometers in either the forward or the rearward directions depending on the cavity eigenenergies. Simulations performed using a finite-difference time-domain (FDTD) method and an analytical model based on a complex dispersion (CD) relation of SPPs (CD model) [51, 52] revealed that for a chirp-induced SPP WP, spectral clipping by an MIM-NC induced spatial shifts.

## 2 Materials and methods

### 2.1 Sample fabrication and experimental setup

**Figure 1:**
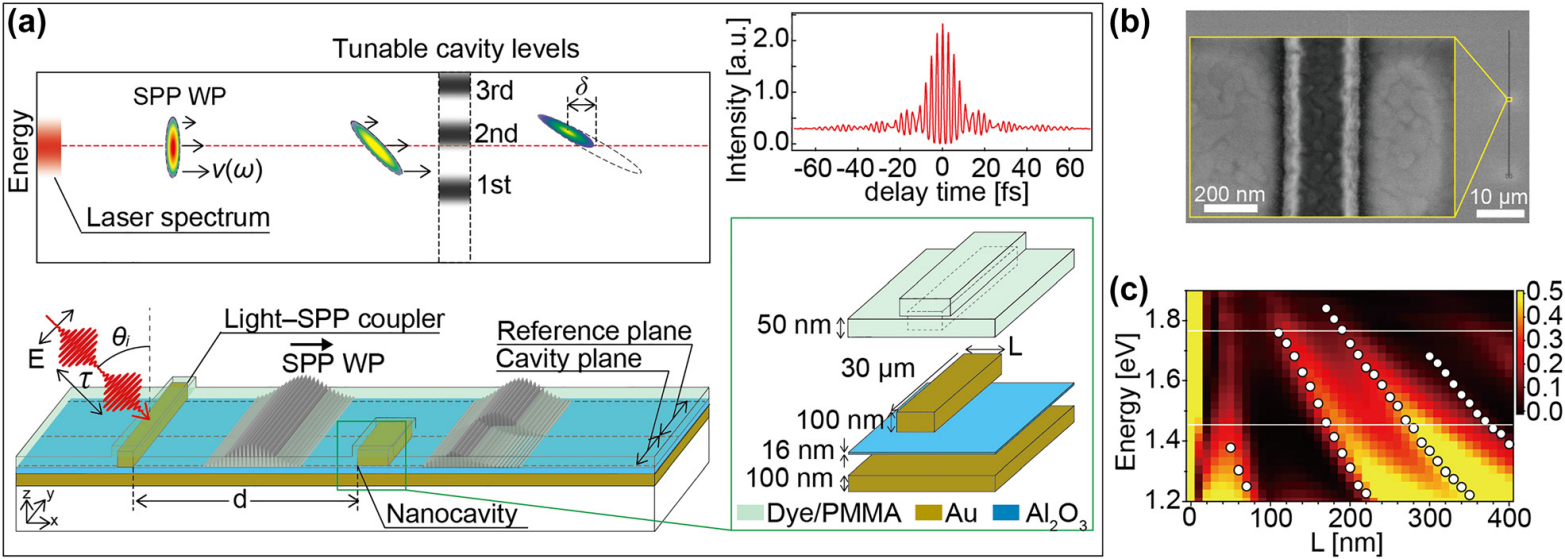
Concept of resonant interaction of SPP WP with nanocavity, overview of the experimental setup, and spectral property of the nanocavity. (a) Schematic of sample containing MIM nanocavity and light–SPP coupler. The sample surface is covered with a dye-doped PMMA thin film. Irradiation with 10 fs laser light launches the SPP WP from the coupler. The SPP WP possesses a spectral distribution comparable to that of the laser light immediately after the excitation. Resonant interaction with an MIM-NC, which has a few discretized levels of the eigenmode, results in a spectrally clipped transmission, leading to a spatial shift ( δ ) in the position of the intensity peak of the WP. A fringe-resolved autocorrelation (FRAC) trace of the fs-laser obtained by placing a thin β -BBO crystal at the sample position is also shown. (b) SEM image of MIM-NC with a length of *L* = 220 nm. (c) Two-dimensional plot of transmittance spectra of SPP plotted as function of length of MIM-NC (*L*; 10–400 nm) and energy (see Supplementary Figure S1 for details). The four sets of solid circles indicate the peak energies of the first- to fourth-order eigenmodes of the MIM-NC. The two white lines indicate the upper and lower spectral limits of the excitation laser.

To study the optical interaction of an SPP WP with an MIM-NC, a 10 fs SPP WP was made to propagate over an Au surface containing an MIM structure. Figure 1(a) shows a schematic of the launching of an SPP WP by the incidence of femtosecond light and the interaction of the WP with an MIM-NC having a few eigenenergies near the spectral range of the SPP. The MIM-NCs were prepared by placing rectangular Au nanoblocks on an Al_2_O_3_ (thickness (*h*) = 16 nm)/Au (*h* = 100 nm) film (Figure 1(a) and (b)). The eigenenergy levels were determined as a function of the MIM-NC structure length *L*, which corresponds to the length of the waveguide for the SPP inside the cavity. The MIM-NCs were fabricated by sequentially changing the length *L* in the range of 50–220 nm. On the same Au surface, another straight MIM structure was placed 40 μm apart from the MIM-NC as a light–SPP coupler. The entire area of the surface was coated with a thin dye-doped PMMA layer to form a two-photon fluorescent layer. The dye (coumarin 343) used for the fluorescent layer has absorption and emission peaks at approximately 440 and 470 nm, respectively. The wavelength of the absorption line is shorter than that of the femtosecond laser; thus, the dye doping does not affect the linear extinction coefficient of PMMA.

### 2.2 Time-resolved two-photon fluorescent microscopy of SPP WP

The light source was a custom-made Ti:sapphire laser oscillator with a pulse duration of 10 fs, a spectral distribution ranging from 680 nm to 900 nm (center wavelength: 810 nm), a repetition rate of 90 MHz, and an average power of 450 mW. Light pulses were generated such that they formed coaxially aligned pump–probe pulse pairs using a Mach–Zehnder interferometer (MZI) [50, 58]. A piezoactuator-driven optical stage was used to control the length difference between the two arms of the MZI with a precision of <20 nm (<70 as) to ensure an interferometric phase correlation between the pump and probe. Then, the formed pulses were loosely focused onto an oval-shaped spot (80 × 60 μm) on the sample surface where both an MIM-NC and a light–SPP coupler were placed. The laser power per pulse on the sample surface was 0.9 μJ/mm^2^. P-polarized, dispersion-compensated pump–probe pulses were used to irradiate the sample at an incident angle ( $\theta_{i}$ ) of 45° from the surface normal. The incident light launched SPP WPs from the coupler in both the positive and negative *x* directions. In addition, the MIM-NC itself worked as a light–SPP coupler. SPP WPs that were excited by a pump pulse on the coupler and MIM-NC interfered with the pump pulse itself immediately after the excitation or with a probe pulse after propagation on the surface for a period determined by the pump–probe delay time ( $\tau_{d}$ ), group velocity of the SPP WP ( $v_{g}^{SPP}$ ), and $\theta_{i}$ . The latter interference evolved as a function of $\tau_{d}$ and included dynamical properties of the SPP WP (see Supplementary Figure S3 for details). The probe pulse also excited SPP WPs as same as the pump pulse, however, they didn’t contribute to the $\tau_{d}$ -dependent interference. Consequently, transient gratings of the surface fields were formed near the coupler and MIM-NC, or on the flat surface area. The latter transient grating represents the spatiotemporal dynamics of the SPP WP [51], [52], [53]. These modulations of the surface fields were imaged as interference beat patterns in micrographs by converting the fields to fluorescence in the PMMA layer on the Au surface through two-photon excitation of dye-molecules. The fluorescent emissions were then detected with 0.5 μm resolution using a long-working-distance objective lens (N.A. 0.55, M Plan Apo SL100X, Mitutoyo) equipped with a band-pass filter (T: 475–495 nm, Semrock) and an electron-multiplying charge-coupled-device camera (Rolera EM-C2, QImaging) [49]. The signal intensity detected by the CCD camera, I , was formulated as a time integral of the fourth power of the total electric field,

(1) $$\begin{array}{l} I\left(x\right)=\overset{\infty }{\underset{-\infty }{\int }}{\left(|E_{SPP}\left(x,t\right)+E_{Light}\left(x,t\right){|}^{2}\right)}^{2}dt, \end{array}$$

where, $E_{SPP}$ and $E_{Light}$ are the electric field of the SPP and the light-induced surface field, respectively.

Time-resolved movies of the SPP WPs were obtained by taking a sequence of fluorescent images while advancing $\tau_{d}$ by incremental steps of π/2 rad of the carrier wave or by 0.68 fs. The beat patterns in the movies, which evolve as a function of $\tau_{d}$ , can be interpreted as spatially resolved second-order cross correlations of the SPP WPs. The dynamical properties of the SPP WPs, such as the group velocity and envelope shape deformation, were determined from the time-resolved images [53]. As discussed later, our investigation focused on a component of SPP WPs launched in the positive *x* direction from the coupler.

### 2.3 SPP WP propagation on a surface equipped with MIM-NC

Because the SPP WPs excited at the coupler replicated the temporal waveform of the light pulse, they initially had a frequency spectrum comparable to that of the light pulse and had the narrowest temporal duration corresponding to the transform limit. Then, the SPP WPs propagated along the surface to interact with the MIM-NC while the spatial width extended and the carrier frequency up-chirped owing to the dispersion relation of the SPPs at the sample surface (Figure 1(a)). The dispersion relation of SPP was determined by the refractive indices of materials that constructing the multi-layered sample structure (see Supplementary Figure S5 for derivations of dispersion curves). This system is similar to a system consisting of an optical cavity coupled with a waveguide [59], [60], [61]. When the SPP WPs reached the MIM-NC, the wave components whose frequencies coincided with the eigenenergy of the cavity were coupled to the eigenmode. Consequently, a large part of the energy of the SPP WP was squeezed into the thin insulator layer of the MIM-NC and the intensity of the field inside the cavity was enhanced. After a propagation through the MIM-NC at a slowed group velocity corresponding to the MIM waveguiding mode, the SPP WP was released from the other end of the MIM-NC [62]. This process constituted a spectrally filtered transmission of an SPP WP through an MIM-NC. Because of the normal-dispersion character of the SPP mode on the Au surface, SPP WP causes up-chirping and thus the head and tail regions of the WP reached to the MIM-NC possesses different carrier frequencies. Therefore, the spectral filtering by MIM-NC leads to a spatial deformation of the SPP WP. Our previous FDTD studies have suggested that if an SPP WP is chirped, the transmitted SPP WP will exhibit a shift ( δ in Figure 1(a)) in terms of the spatial coordinate of the intensity peak compared to an SPP WP that propagates on a flat surface. When the SPP WP is up-chirped and the eigenenergy of the MIM-NC matches the upper (lower) part of the WP spectrum, the spectral clipping by the MIM-NC causes a shift ( δ ) in the rearward (forward) direction. The amount of δ is closely related to the transmittance spectrum and to the amount and the sign of chirp in the SPP WP.

The transmittance spectra of an SPP through an MIM-NC were evaluated using the FDTD method for the cavity length (*L*) range of 10–400 nm and are plotted in Figure 1(c) together with the first- to fourth-order eigenmodes of the MIM-NC (open circles) (see Supplementary Figure S1 for details) [36]. Overall, the transmittance spectra followed the trends of the eigenmodes of the cavity. More specifically, the spectra exhibited asymmetric line shapes, with the peak energy being slightly shifted from the eigenmodes. These features are interpreted as the Fano-resonance line shape that arose as a consequence of the interference between the discretized eigenmodes of the MIM-NC and the continuous SPP mode on the surface of the MIM nanoblock structure [63], [64], [65], [66]. While the transmittance reached considerably large values (∼0.5) at the peaks, it decreased considerably (∼0.1) for *L* ≈ 100 nm, corresponding to a valley between the first- and second-order resonances.

## 3 Results

### 3.1 Deformation of SPP WP by transmission through MIM-NC

**Figure 2:**
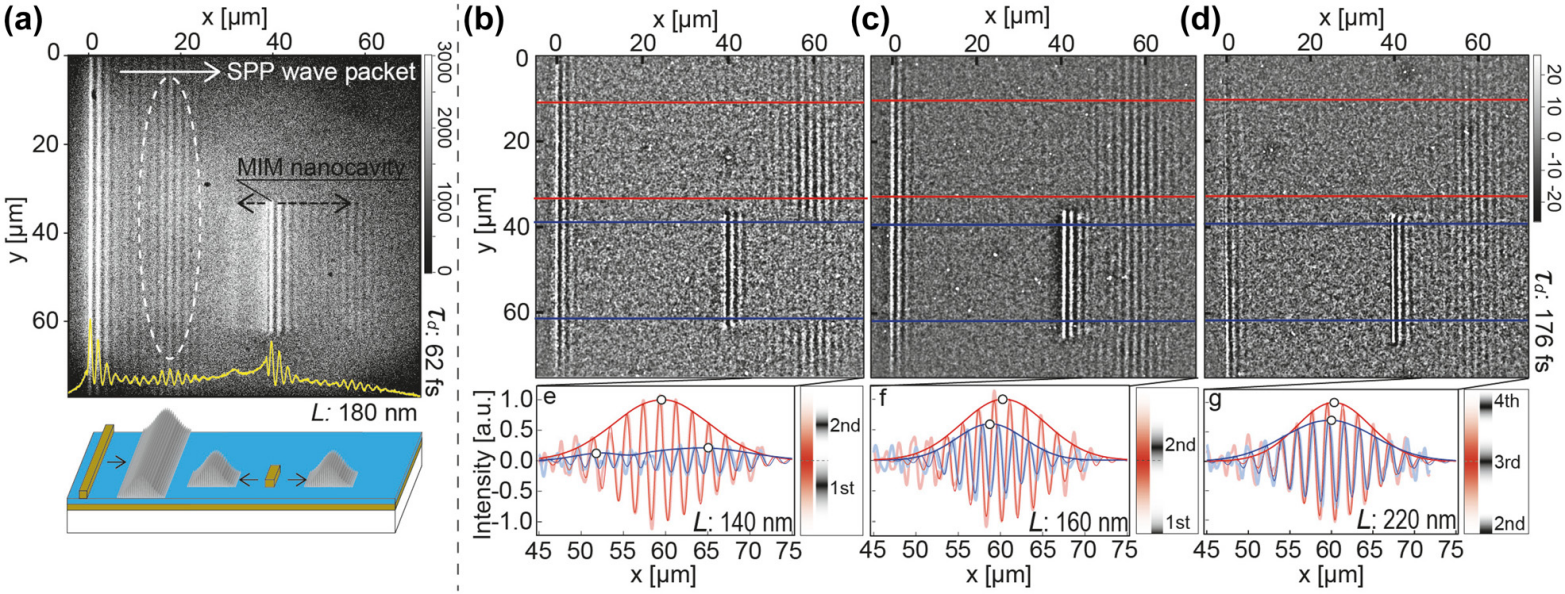
Fluorescence images of SPP WPs. (a) Selected frame of time-resolved two-photon fluorescence movie ( $\tau_{d}$ = 62 fs) and cross section taken along *x-*direction (yellow line). (b)–(d) Time-resolved micrographs of SPP WPs transmitted through MIM-NCs with length *L* = (b) 140 nm, (c) 160 nm, and (d) 220 nm for ( $\tau_{d}$ ) = 176 fs. The beat patterns, which revealed the spatial distributions of the WP, were constructed according to the interference between an SPP WP and a probe pulse and were extracted by removing background and delay-independent intensities (see Supplementary Figure S4 for details). The red and blue lines indicate the reference and cavity planes, respectively. The cross sections of the beat patterns in the reference and cavity planes in (b), (c), and (d) were prepared by addition-averaging the images along the *y* direction and are shown by the thick light-red and light-blue lines in (e), (f), and (g), respectively. The least-square fits and envelopes of the patterns are shown by thin dark-red and dark-blue lines. The cross sections in the reference planes (*L* = 140, 160, and 220 nm) and those in the cavity planes with *L* = 160 and 220 nm were fitted by a single sine-Gaussian function, but the cross section in the cavity plane with *L* = 140 nm was fitted by the sum of two sine-Gaussian functions. The energy distributions of a spectrum of the SPP WP and the eigenmodes of an MIM-NC are schematically shown in the insets of (e)–(g). The MIM-NCs with *L* = 140, 160, and 220 nm provide interactions represented by (e) a valley of two resonance modes, (f) slightly detuned resonance to a higher energy side, and (g) an almost on-resonance condition, respectively (see Supplementary Movie 1 for the full-frame movie for the MIM-NC with *L* = 180 nm). The exposure times for (a) was 60 s and for (b–d) were 900 s.

Figure 2(a) shows a frame of the time-resolved fluorescence movie taken for a pump–probe delay time of $\tau_{d}=$ 62 fs (see Supplementary Movie 1 for the full-frame movie). The micrograph shows noticeable beat patterns. The strong oscillations observed near the coupler ( x=0 ) and MIM-NC ( x= 40 μm) are caused by the interference between the pump-excited SPP WP and the pump pulse (self-interference), or between the probe-excited SPP WP and the probe pulse that also constructs the self-interference. Other oscillatory patterns appearing at x ≈ 20 and 60 μm represent interference between the pump-excited SPP WPs, respectively, launched from the coupler and MIM-NC, and the probe pulse (pump–probe interference). The beat patterns arising from the pump–probe interference moved in the positive x direction as $\tau_{d}$ advanced, reflecting the propagation of SPP WPs. The $\tau_{d}$ -dependent movement and changes in the spatial shape of the beat pattern that evolved from the coupler were traced. It should be noted that another SPP WP was launched in the negative x direction from the MIM-NC. It was imaged as a faint one-dimensional increment of the intensity at x ≈ 30 μm, as shown in Figure 2(a). The spatial wavelength of the beat pattern, $\lambda_{beat}$ , was determined by the reciprocal of the difference in the wavenumbers between the SPP and the in-plane component of the incident light as

(2) $$\begin{array}{l} \lambda_{beat}=\frac{2\pi }{\left|k_{SPP}-k_{0}\;\sin\;\theta_{i}\right|}, \end{array}$$

where $k_{SPP}$ and $k_{0}$ are the wavenumbers of the SPP WP and incident light, respectively. The $\lambda_{beat}$ lengths were evaluated as 1.7 and 0.4 μm for the SPPs propagating in the positive and negative x directions, respectively. The length of 0.4 μm was finer than the resolution of the microscope, and thus, it was not pursued further in this study.

**Supplementary Movie 1:** 

The SPP WP launched from the coupler underwent a resonant interaction with an MIM-NC, passed through it, and continued to propagate on the metal surface at the far side of the cavity. The deformed spatial shapes of the SPP WPs were determined by analyzing the beat patterns in this area. For detail examination of the SPP WP, the oscillatory waveform constructed by the pump–probe interference was extracted by the following method: The constant phase of the pump-probe interference beat moves by $\lambda_{beat}$ while $\tau_{d}$ increases by 2 π rad (2.7 fs), which corresponds to a cycle of the carrier wave of the laser. Therefore, when time-resolved images, $I\left(\tau_{d}\right)$ , are addition averaged over the delay range of ( $\tau_{d}-2.7/2\text{ fs})∼(\tau_{d}+2.7/2\;fs)$ , the resulted image flattens the pump-probe oscillatory beat and only leaves the delay-independent components. Assuming the time-resolved images are taken with an interval of the delay $\Delta \tau_{d}=\left(2.7/m\right)$ fs where m is an integer, the phase-averaged image for $\tau_{d}$ , $\bar{I}\left(\tau_{d}\right)$ , is obtained as

(3) $$\bar{I}\left(\tau_{d}\right)=\frac{1}{m}\overset{m}{\underset{i=1}{\sum }}I\left(\tau_{d}-\frac{2.7}{2}+\left(i-1\right)\cdot \Delta \tau_{d}\right).$$

This procedure practically works well because the change in the special envelope of the pump-probe beat during an increment of $\tau_{d}$ by 2.7 fs is small. Then, a background-free image of the pump-probe beat at $\tau_{d}$ , $I_{WP}\left(\tau_{d}\right)$ , which is sensitive to the delay and directly reflecting the waveform of the SPP WP, is obtained by subtracting $\bar{I}\left(\tau_{d}\right)$ from $I\left(\tau_{d}\right)$ ,

(4) $$I_{WP}\left(\tau_{d}\right)=I\left(\tau_{d}\right)-\bar{I}\left(\tau_{d}\right).$$

In this study, we minimized the number m to avoid the breaching of the fluorescence layer during the laser irradiation. When $\Delta \tau_{d}$ is set to π rad (1.35 fs), m is reduced to 2. Equation (3) can be modified to

(5) $$\bar{I}\left(\bar{\tau_{d}}\right)=\frac{1}{2}\left(I\left(\tau_{d1}\right)+I\left(\tau_{d2}\right)\right),$$

where, $\bar{\tau_{d}}=\left(\tau_{d1}+\tau_{d2}\right)/2$ , $\tau_{d1}=\left(n-0.25\right)\times$ 2.7 fs and $\tau_{d2}=\left(n+0.25\right)\times$ 2.7 fs where n is an integer. Then, the difference between $I\left(\tau_{d1}\right)$ and $I\left(\tau_{d2}\right)$ is

$$I\left(\tau_{d1}\right)-I\left(\tau_{d2}\right)=\left(I_{WP}\left(\tau_{d1}\right)+\bar{I}\left(\bar{\tau_{d}}\right)\right)-\left(I_{WP}\left(\tau_{d2}\right)+\bar{I}\left(\bar{\tau_{d}}\right)\right)$$

(6) $$=I_{WP}\left(\tau_{d1}\right)-I_{WP}\left(\tau_{d2}\right)\approx 2I_{WP}\left(\tau_{d1}\right)\equiv 2I_{WP}\left(\bar{\tau_{d}}\right).$$

As similar to $I_{WP}\left(\tau_{d}\right)$ in Eq. (4), $I_{WP}\left(\bar{\tau_{d}}\right)$ again gives a background-free image of the pump-probe beat at $\bar{\tau_{d}}$ (see Supplementary Figure S4 for a specific example of the image processing).

Figure 2(b)–(d) shows the differential images taken at $\bar{\tau_{d}}=$ 176 fs for the MIM-NCs with lengths L of 140, 160, and 220 nm, respectively. With this delay time, the SPP WPs passed through the MIM-NCs and reached x= 60 μm. The cross sections of the beat patterns shown in Figure 2(b)–(d) were prepared by addition-averaging the images along the y direction over the areas indicated by two blue solid lines (cavity planes) and are shown in Figure 2(e)–(g), respectively. For a controlled comparison, cross sections were also obtained for the flat regions indicated by red solid lines (reference planes) in a similar manner. The beat patterns in the reference planes showed almost identical features for the cavities with *L* = 140, 160, and 220 nm, whereas those in the cavity planes varied sensitively to *L*. The peak intensity in the cavity plane normalized to that in the reference plane largely decreased (∼0.2) for *L* = 140 nm but remained considerably large (>0.5) for *L* = 160 and 220 nm. The WPs were also associated with changes in envelope shapes. For the 220 nm cavity, the beat pattern in the cavity plane was well fitted by a single Gaussian-modulated sinusoidal wave function, and the peak position and width of the envelope for the cavity plane were comparable to those for the reference plane. A single sine-Gaussian wave shape was also found for the 160 nm cavity; however, the envelope peak for the cavity plane showed a distinct backward position shift with respect to that for the reference plane. For the 140 nm cavity, the wave was reasonably fitted by the superposition of two sine-Gaussian functions, and the envelope peaks for the cavity plane advanced by 5 μm and retarded by 7 μm with respect to the peak for the reference plane.

The variations in the spatial deformations of the SPP WPs were closely related to the levels of the eigenenergies of the MIM-NC, which sensitively changed as a function of *L*. The MIM-NC was a plasmonic Fabry–Pérot resonator constructed by cutting an MIM plasmonic waveguide to a length that was approximately equal to multiple halves of the SPP wavelength in the MIM, i.e., $L\approx \zeta \lambda_{MIM}/2$ , where ζ is the resonance order [38, 40]. The SPP wavelength in an MIM structure is considerably compressed compared to that on a metal surface; in the case of an Au/Al_2_O_3_/Au structure with an Al_2_O_3_ layer thickness of 16 nm, $\lambda_{MIM}$ was estimated as 206 nm for an excitation light wavelength of 810 nm. Therefore, a change in L of only a few tens of nanometers considerably modified the distribution of eigenenergies. The relationship between the spectral distribution of the SPP WPs and the eigenenergies of the MIM-NCs is schematically shown in the insets of Figure 2(e)–(g). For L = 140 nm, a major part of the WP spectrum did not overlap with the eigenmodes (off-resonance condition, Figure 2(e)). Only the lower and higher energy ends of the spectral distribution overlapped with the first and second eigenmodes of the MIM-NC, respectively. Consequently, the transmittance of the SPP WP was maintained at a low level, and the envelope shape split into two peaks. When L was extended from 140 nm to 160 nm, the eigenenergy of the second mode decreased and constituted a larger overlap with the WP spectrum; however, the center energy of the second mode was still higher than that of the spectrum peak (Figure 2(f)). This detuned resonance led to a larger transmission of the SPP WP and a delay in the peak position. A further extension of L to 220 nm led to the resonance of the eigenenergy of the third mode with the WP spectrum, resulting in a large transmission of the SPP WP (Figure 2(g)). Under such an on-resonance condition, the peak shift was maintained at an almost negligible level.

### 3.2 Sequence of time-resolved movie frames of SPP WP

**Figure 3:**
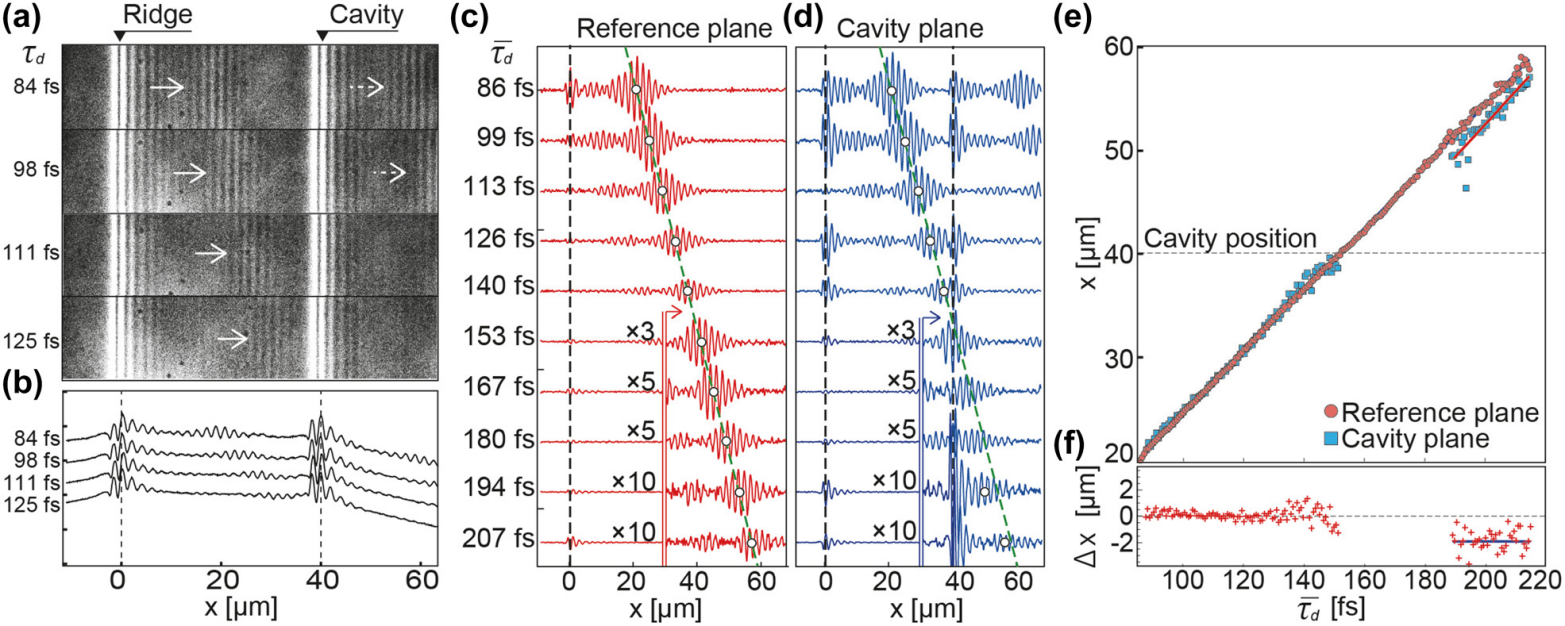
Sequences of time-resolved fluorescence images of SPP WP. (a) Selected frames of time-resolved two-photon fluorescence movie. The cross sections of the beat patterns in (a) were prepared by addition-averaging the images along the *y* direction and are shown in (b). Cross sections of time-resolved differential micrographs taken from (c) reference plane and (d) cavity plane containing MIM-NC with *L* = 160 nm. The range of the delay time was $\bar{\tau_{d}}$ = 86–207 fs with an interval of 13.5 fs or 5 × 2 π rad of the carrier wave of the excitation laser. The open circles indicate the coordinates of the intensity maxima of the beat patterns. The green dotted line in (c), indicating a constant advancement of an SPP WP, is duplicated and plotted in (d). (e) Coordinates of intensity peaks of beat patterns taken from reference plane (red circles) and cavity plane (blue squares) plotted as function of average delay time. The intensity maxima in the cavity plane exhibited a homogeneous negative shift compared to those in the reference plane over the region where isolated WP forms were recovered ( x≥ 50 μm). The amounts of shifts ( Δx ) are shown in (f). The exposure times for microscopy images ranged from 5 to 40 s depending on the delay time. The intensity of each image was normalized by the exposure time.

The spatial shifts in the intensity peaks of the SPP WPs were further investigated by examining a sequence of time-resolved images from the TR-2PFM movie. Figure 3(a) shows selected frames from the movie for $\tau_{d}$ = 84–125 fs; in this case, the pump–probe interference beat of the SPP WP was located between the coupler and MIM-NC. The length of the MIM-NC was L = 160 nm. The cross sections of these frames (Figure 3(b)) indicate the progression of the beat pattern; however, it was difficult to continue tracking at x≥ 40 μm because of the strong $\tau_{d}$ -independent self-interference beat near the MIM-NC. To extract the $\tau_{d}$ -dependent dynamics of the pump–probe beat, differential images were taken for $\bar{\tau_{d}}$ = 86–207 fs. The cross sections of these images in the reference and cavity planes are shown in Figure 3(c) and (d), respectively. The spatial shape of the pump–probe interference beat imitated the waveform of the pump-excited SPP WP (see Supplementary Figures S3 and S4). The beat pattern in the reference plane showed that the intensity peak (open circles in Figure 3(c)) propagated at a constant rate, whereas the beat pattern in the cavity plane (Figure 3(d)) showed a rearward peak shift compared to that in the reference plane (green dotted line) after transmission through the MIM-NC placed at *x* = 40 μm. In the following delay times, the beat pattern in the cavity plane continued to propagate at a constant rate while maintaining the delay amount of the peak shift. The coordinates of the intensity peaks of the beat patterns are plotted in Figure 3(e) for $\bar{\tau_{d}}$ = 86–214 fs with an interval of 0.68 fs (0.25 × 2 π rad). The two sets of peaks in the reference and cavity planes overlapped each other for x < 40 μm, and then, the peaks in the cavity plane exhibited a constant negative shift of −1.83 μm after passing through the MIM-NC (Figure 3(f)). The group velocities of the SPP WPs in both the planes were evaluated as $v_{g}^{SPP}$ = 1.75 × 10^8^ m/s (0.58*c*, where *c* is the speed of light in vacuum), which agreed well with the group velocity derived from the first derivative of the dispersion curve of the SPP mode at the PMMA/Al_2_O_3_-layer-coated Au surface.

## 4 Discussion

### 4.1 Modeling of WP deformation through resonant interaction with MIM-NC

The TR-2PFM imaging of SPP WPs revealed how the waveform and intensity of the transmitted SPP WP changed significantly according to the overlap between the spectrum of the WP and the eigenenergy of the MIM-NC. When the eigenenergy was detuned from the center of the spectral distribution, the transmitted WP showed a shift in the peak intensity position compared to the case continuing the propagation on a flat surface. This result is qualitatively consistent with the recent FDTD simulation [36] and another time-resolved measurement study of SPP wave scattering by a nanohole [67]. The waveform deformation and peak shift observed in a transmitted SPP WP are attributed to the clipping of a chirped SPP WP in the temporal-frequency domain induced by an MIM-NC. Because of the normally dispersed character of the SPP mode, the instantaneous carrier frequency of an SPP WP gradually becomes up-chirped during propagation on an Au surface. Whereas the intensity peak continuously propagates at a group velocity $v_{g}^{SPP}$ , the wave components of higher (lower) frequencies proceed at slower (faster) speeds. A frequency chirp in the time domain is correlated with the wavenumber chirp in the spatial domain. When an SPP WP passes an MIM-NC that possesses a resonance frequency coinciding with the higher (lower) part of the spectral bandwidth, the peak position of the transmitted WP shifts rearward (forward). If two different resonances contribute, the WP is expected to have two separate peaks, as shown in Figure 2(e).

To obtain more detailed insights into the deformation of the WPs, model calculations were performed based on the CD relations of SPPs (CD model; see Supplementary Figures S2 and S3 for formulations and Movie 2 for simulated time-resolved movie.) [51, 52]. In this model, the temporal waveform of an SPP WP at position *x*, $E_{SPP}\left(x,t\right)$ , was retrieved from polar Fourier expansion coefficients. The Fourier transform of the SPP WP at position x=0 , $E_{SPP}\left(0,t\right)$ is

(7) $$F\left(0,\omega \right)=\overset{+\infty }{\underset{-\infty }{\int }}E_{SPP}\left(0,t\right){\text{e}}^{-i\omega t}dt.$$

**Supplementary Movie 2:** 

The $E_{SPP}\left(0,t\right)$ was assumed to have a same waveform as the light field at x=0 ( $E_{Light}\left(0,t\right)$ ) except for an initial phase difference between the SPP and the light pulse. The modulus and the argument of the complex Fourier coefficient for the frequency $\omega_{i}$ represented in polar coordinate provide the initial amplitude, $R\left(0,\omega_{i}\right),$ and phase, $\phi \left(0,\omega_{i}\right)$ , of the $E_{SPP}\left(0,t\right)$ , respectively. The coefficients of the waveform at position x were evaluated by using the CD ( $k_{SPP}\left(\omega \right)=k_{SPP}^{\prime }\left(\omega \right)+ik_{SPP}^{\prime \prime }\left(\omega \right)$ ) of the SPP as

(8) $$\begin{array}{l} R\left(x,\omega_{i}\right)=R\left(0,\omega_{i}\right)\cdot \exp\left(-k_{SPP}^{\prime \prime }\left(\omega_{i}\right)\cdot \Delta x\right), \end{array}$$

(9) $$\begin{array}{l} \phi \left(x,\omega_{i}\right)=\phi \left(0,\omega_{i}\right)+k_{SPP}^{\prime }\left(\omega_{i}\right)\cdot \Delta x. \end{array}$$

Here, $k_{SPP}^{\prime }$ and $k_{SPP}^{\prime \prime }$ are the real and imaginary parts of the complex wavenumber of the SPP, respectively [36, 68]. The waveform of the $E_{SPP}\left(x,t\right)$ was obtained by the inverse Fourier transform as

(10) $$E_{SPP}\left(x,t\right)=\frac{1}{2\pi }\overset{+\infty }{\underset{-\infty }{\int }}R\left(x,\omega \right){\text{e}}^{i\left(\omega t+\phi \left(x,\omega \right)\right)}d\omega .$$

The CDs of the SPP modes for the PMMA/Al_2_O_3_/Au surface and Au/Al_2_O_3_/Au waveguiding structure were independently prepared (see Supplementary Figure S5 for derivations of the CDs). The resonant interactions of the SPP WPs with the MIM-NCs were modeled by considering (1) the resonant and/or off-resonant excitations of the multiple eigenmodes of the MIM-NC associated with a phase shift in the SPP WP determined as the sum of the responses to each of the eigenmodes (1st–5th order), (2) phase accumulations in $\phi \left(x,\omega_{i}\right)$ over the cavity length L according to the wavenumber of the SPP inside the MIM-NC determined by the dispersion relation of the Au/Al_2_O_3_/Au waveguiding structure, and (3) the spectral filtering according to the transmittance spectra of the MIM-NCs (Figure 1(c)) (see Supplementary Figure S2 for whole calculations of CD model).

The waveform of $E_{Light}\left(0,t\right)$ was determined by invers Fourier transform of a spectrum of the laser pulse measured by a spectrometer (Ocean Optics, HR4000). The wavefront of a light pulse moves on the sample at a speed of $c/\sin\;\theta_{i}$ . Therefore, the light field at position x , $E_{Light}\left(x,t\right)$ , was determined as

(11) $$E_{Light}\left(x,t\right)=E_{Light}\left(0,t-\frac{x\;\sin\;\theta_{i}}{c}\right).$$

The intensity of the emission of fluorescence induced through two-photon excitations was evaluated by substituting Eqs. (10) and (11) to Eq. (1).

As mentioned above, beat patterns in micrographs comprised of the pomp-probe interferences imitated the spatial shapes of SPP WPs, although the spatial widths of the envelopes were widened than that of the actual SPP WPs. This broadening happens because the excitation light was incident at an oblique angle. For the same reason, an actual spatial separation ( δ ) between SPP WPs at the reference plane and at the cavity plane was detected as an expanded spatial shift ( Δx ) in the micrograph. The relation between Δx and δ is written as

(12) $$\Delta x=\delta {\left(1-v_{g}^{SPP}\frac{\sin\;\theta_{i}}{c}\right)}^{-1}.$$

For the experimental condition used here, Eq. (12) provides a relation Δx=1.7δ .

**Figure 4:**
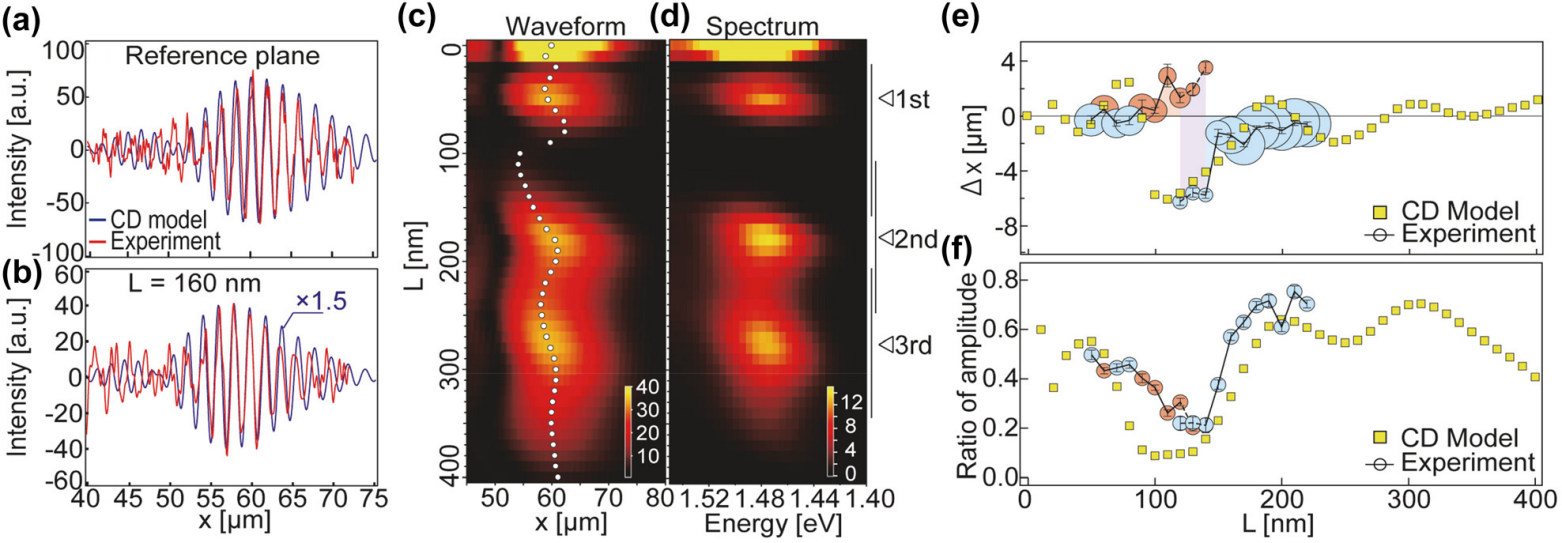
Peak shift, spectral modulation, and amplitude attenuation of SPP WP examined by CD model and experiment. The beat patterns calculated by the CD model for (a) the reference plane and (b) the cavity plane containing an MIM-NC with *L* = 160 nm (blue lines) and their corresponding experimental results obtained from the time-resolved micrographs (red lines) are shown. The average delay time ( $\bar{\tau_{d}}$ ) was 185 fs, at which the intensity peak of a beat pattern in the reference plane reached *x* = 60 μm in both the calculations and experiments. (c) Two-dimensional plots of envelope of beat pattern calculated by CD model as function of length of cavity (*L*) and *x*. The white circles indicate the peak positions of each envelope. (d) Two-dimensional plots of spectra of transmitted SPP WPs calculated by CD model as function of *L* and energy. (e) Extent of peak shift of beat pattern in cavity plane compared with that in reference plane. The yellow squares and colored circles show the peak shifts determined by the CD model and experiment, respectively. The red and blue circles indicate the forward and rearward peak shifts, respectively. The diameter of a circle indicates the amplitude of a beat pattern. For *L* = 120–140 nm (purple-colored region), peak shifts are indicated by two circles reflecting the double-peak feature (Figure 2(d)) of an experimental beat pattern. (f) Ratio of maximum amplitude of beat pattern in cavity plane to that in reference plane plotted as function of *L*. The yellow squares and colored circles show the results of the CD model and experiment, respectively.

Figure 4(a) and (b) shows the beat patterns calculated by the CD model for the reference plane and the cavity plane containing an MIM-NC with L = 160 nm, respectively; the patterns were superimposed on the corresponding cross sections of the time-resolved micrographs. The delay time $\bar{\tau_{d}}$ was 185 fs. The amplitude for the CD model in Figure 4(b) was multiplied by a factor of 1.5 to compensate for the inhomogeneity of the light field on the sample surface. The CD model results agreed well with the experimental results, and the rearward shift of the peak associated with an asymmetric deformation of the envelope of the transmitted SPP WP was reasonably reproduced. The envelope shapes of the beat patterns calculated for the delay time $\tau_{d}$ = 185 fs and MIM-NC length *L* = 0–400 nm are shown in Figure 4(c). For *L* values greater than 20 nm, the plot exhibited three elliptically shaped strength distributions, which corresponded to the first- to third-order resonance modes of the MIM-NCs. Each distribution was slanted off the horizontal axis. In each ellipse, the peak position (open circles in Figure 4(c)) had the maximum rearward shift at the shorter end of the length *L* and gradually shifted forward as *L* increased. The relationship between the peak position and *L* was closely correlated with the relationship between the peak energy in the WP spectrum and *L*. Figure 4(d) shows the calculated Fourier spectra of the SPP WPs at *x* = 50 μm after transmission through the MIM-NC with *L* = 0–400 nm. Overall, the configuration shown in Figure 4(d) is very similar to that shown in Figure 4(c). The peak energy was blue (red)-shifted for an SPP WP with a retarded (forwarded) peak shift; this observation is consistent with the mechanism of spectral clipping of an up-chirped SPP WP by an MIM-NC.

As shown in Figure 4(e) and (f), the CD-model-calculated peak shifts ( Δx ) and amplitudes of the beat pattern in the cavity plane showed reasonable consistency with the experimental results. Time-resolved micrographs for MIM-NC lengths of *L* = 50–220 nm and a delay of $\bar{\tau_{d}}$ = 185 fs showed that Δx can be tuned in either the forward (red circles in Figure 4(e)) or the backward (blue circles) direction in the range of a few microns. Forward and backward shifts were obtained for cavity lengths of *L* = 90–110 and 150–220 nm, respectively. At a boundary (*L* = 120–140 nm, purple-colored area in Figure 4(e)), the amplitudes of the transmitted SPP WPs were largely attenuated (Figure 4(e)), and the envelopes deformed to such an extent that they were reasonably fitted by the sum of two Gaussian curves.

Because the coherence lifetimes of MIM-NCs are limited to several femtoseconds, MIM-NCs possess wide linewidths and behave as low-finesse Fabry–Pérot etalons. SPP WPs sustain spectral widths that are sufficiently wide to form a WP with femtosecond time durations even after transmission through an MIM-NC. As shown in Figure 4(b) and (c), the spectral clippings by MIM-NCs did not elongate the width of the WPs or induce complicated phase retardations in the WPs. Rather, the width of the SPP WPs could be narrowed by transmission through an MIM-NC if the incident WP had a large chirp. The slow group velocity of the MIM waveguiding mode compared to that of the SPP on the metal surface induced a retardation of the SPP WP, but the retardation was only marginal; for the MIM-NC with *L* = 160 nm, the retardation effect was estimated to be only Δx=−0.25 μm. Accordingly, the tunable range of the peak shift was mostly dominated by the spatial width of the chirped incident SPP WP.

### 4.2 External control of peak shift of WP using chirped light pulse

The control of the peak shifts of the transmitted SPP WPs can be regarded as an application of spatiotemporal coupling (STC) [25, 26]. In the abovementioned case, the STC was simply an up-chirping of the carrier wave of an SPP WP that possessed longer (shorter) wavelengths in the head (tail) region. The intensity peaks of the SPP WPs were controlled by tuning the eigenenergy of the MIM-NC such that a portion of the carrier wave was clipped, with the instantaneous frequency matching the eigenenergy. Here, another strategy for controlling the peak shifts is discussed wherein the chirp applied to the excitation light pulses is externally controlled. CD model calculations were performed to estimate the amount of shift Δx by applying under- or overcompensated chirps to the excitation light pulse. The phase ( $\phi_{c}$ ) distribution of a chirped pulse at x = 0 is given as $\phi_{c}\left(0,\omega_{i}\right)=\phi \left(0,\omega_{i}\right)+\phi_{g}\left(\omega_{i}\right)$ , where $\phi_{g}\left(\omega_{i}\right)$ is the extra phase added to the phases of the transform-limited pulse. For the case of a light pulse that has passed through a dispersive material, $\phi_{g}$ is given as $\phi_{g}\left(\omega_{i}\right)=\left(\omega_{i}/c\right)\cdot n_{g}\left(\omega_{i}\right)g$ , where $n_{g}$ is the refractive index and g is the effective thickness of the material. Practically, g can be manipulated in the range of negative to positive values by combining optical elements which respectively provide negative and positive dispersions. For example, a combination of chirped mirror pairs and a glass wedge pair, often used as dispersion compensation mechanism for femtosecond laser pulses, can serve this purpose. A condition of zero effective thickness of the dispersive material ( g=0 ) is achieved by balancing the amount of negative dispersion by the chirped mirror pair with the amount of positive dispersion by the glass wedge pair. If the thickness of the glass wedge is reduced (increased) from this condition, a negative (positive) g is provided.

**Figure 5:**
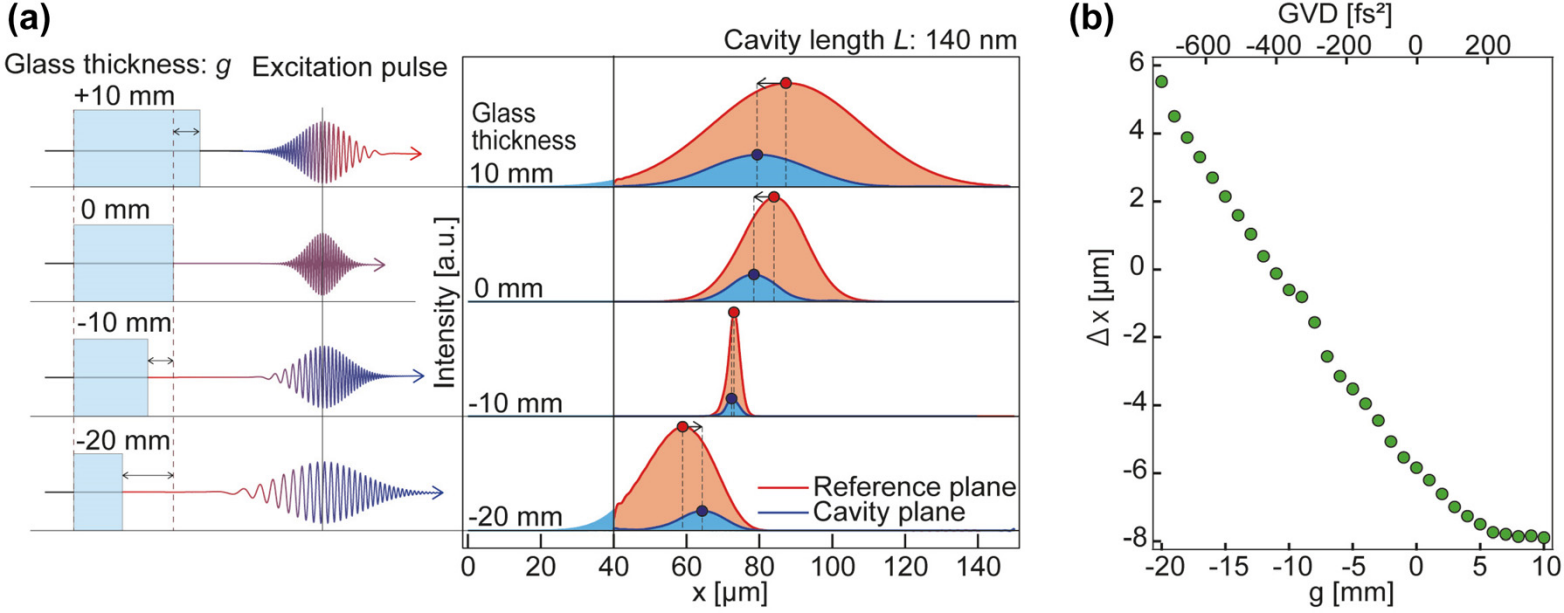
Control of peak shift of SPP WP by the chirp-induced excitation pulse. (a) Envelope shape of SPP WP calculated by CD model for reference plane (red plots) and cavity plane (blue plots) containing MIM-NC with *L* = 140 nm under several chirped pulse conditions. The red and blue circles indicate the coordinates of the intensity maxima of the SPP WPs in the reference and cavity planes, respectively. The temporal waveforms of a chirp-induced excitation pulse are schematically shown. The group velocity dispersion (GVD) of each pulse corresponds to the amount of chirp caused by the transmission of the silica glass with effective thickness *g* = −20 to 10 mm. (b) Extent of peak shift (∆x) of SPP WP after transmission through MIM-NC as function of thickness of silica glass or GVD applied to the excitation pulse (see Supplementary S2 for details).

In conducting the CD model calculations, we assumed fused silica glass as the material [69]. The length of the MIM-NC was assumed to be *L* = 140 nm; the MIM-NC had a second-order resonance at 1.7 eV and was placed at x = 40 μm. This eigenenergy matched a higher-energy region of the spectrum of a 10 fs, 810 nm Gaussian-shaped excitation pulse. Figure 5(a) shows schematic waveforms of the chirped excitation pulses for several glass thicknesses (left side of Figure 5(a)) and the CD model calculations of spatial envelope shapes of the SPP WPs 500 fs after excitation (right side of Figure 5(b)). For a glass thickness of g=0 mm, the peak of the SPP WP in the cavity plane was retarded compared to that in the reference plane, as was expected for the incidence of an up-chirped SPP WP on an MIM-NC with the eigenenergy detuned to the higher side. The rearward shift increased as g increased. However, for negative glass thicknesses, the amount of shift decreased and reached ∼0 for a thickness of g=−10 mm, which corresponded to a group velocity dispersion (GVD) of −360 fs^2^. This null shift was realized because the negative GVD compensated for the positive dispersion of the SPP on the Au surface. For greater negative g values, the SPP WPs maintained down-chirps when they reached the MIM-NC, resulting in the forward shifts of the intensity peaks. Although the center frequency of SPP WP was shifted by passing through the MIM-NC, transmitted WPs maintained the spatial shapes and widths of the envelopes comparable to those of the reference WPs. The peak shifts calculated for g= −20 to 10 mm are plotted in Figure 5(b). Continuous control over the spatial shift of the transmitted SPP WP was achieved in the range of −8 to 5 μm.

It may be useful to mention that the spatial peak shift of SPP WP would arise an apparent anomalous group velocity of the WP in MIM-NC. In MIM waveguides, SPP waves propagate at their intrinsic group velocity defined by the first derivative of the dispersion curve of the waveguiding mode as $v_{g}^{MIM}\left(\omega_{i}\right)={\partial \omega /\partial k_{MIM}|}_{\omega =\omega_{i}}$ , where $k_{MIM}$ is the wavenumber of the SPP in the MIM waveguide. The $v_{g}^{MIM}$ is evaluated as $9.1\times 10^{7}$ m/s (0.30*c*) for the SPP mode excited by the 810 nm-light in the Au/Al_2_O_3_ (*h* = 16 nm)/Au structure. If we assume the SPP WPs propagate through the MIM-NCs with the same group velocity as $v_{g}^{MIM}$ , the peak shift will be $\delta =\left(1-v_{g}^{SPP}/v_{g}^{MIM}\right)L$ . By using this relation, the spatial shifts in TR-2PFM micrographs for the range of the L of 50–220 nm is estimated to be Δx= −0.08–−0.35 μm. Definitely, the observed shifts (Figure 4(e)) amounted much larger, and even positive shifts were realized. These results strongly suggests that the utilization of the resonant interaction of nanocavities and the chirp of WPs will allow for more flexible control of the wavefront of SPP WPs in two-dimensional devices. The range of the available shift is determined by the chirp-induced spatial spreading of the WP. Our experimental results showed the dispersion relation of SPP on metal surfaces could cause the chirp of WP large enough to generate the peak shift in micrometer-range. Moreover, as demonstrated by the CD model calculations (Figure 5(a) and (b)), externally manipulated chirp of the excitation light can also contribute this role. If we assume that the peak shifts of SPP WPs in micrographs are due to the change in the apparent group velocity ( $v_{g}^{ap}$ ) during the transmission through the MIM-NC, the relation between $v_{g}^{ap}$ and δ would be written as $v_{g}^{ap}=v_{g}^{SPP}\cdot L/\left(L-\delta \right)$ [2]. The shift of Δx= + 6–−8 μm evaluated by the CD model for the L = 140 nm cavity (Figure 5(b)) leads the variation of the apparent group velocity index ( $c/v_{g}^{ap}$ ) in the range of about −40–+60.

## 5 Conclusions

In this study, the spatiotemporal dynamics of an SPP WP interacting with an MIM-NC possessing discretized eigenenergies near the spectral distribution range of the WP were investigated using time-resolved microscopy and numerical calculations. The spatial shape and the motion of an SPP WP launched on an Au surface by an irradiation of 10 fs, 810 nm laser pulse were imaged with 10 fs-temporal and 0.5 μm spatial resolutions. A small portion of the energy of surface electromagnetic fields was up-converted to fluorescence emissions through two-photon excitations of a thin dye-doped PMMA layer coated on the surface. Time-resolved two-photon fluorescence microscopy (TR-2PFM) revealed spatial shifts in the peak position of the WP after it passed through the MIM-NC. The transmittance spectra of an SPP WP passing through an MIM-NC were analyzed by FDTD simulations and the waveforms and spectra of the SPP WP were calculated by the CD model. The analysis results showed that the peak shifts were induced by the spatiotemporal clipping of a chirped SPP WP caused by the MIM-NC. The peak shift can be controlled to within several micrometers in either the positive or the negative direction by adjusting the eigenenergy of the MIM-NC or the chirp of an excitation pulse. The controllability of the SPP WP demonstrated here is expected to provide useful insights into the development of novel optical devices based on the complex control of light waves using optical nanostructures.

## 6 Methods

### 6.1 Sample fabrication

The sample was prepared as follows. First, an Au layer with a thickness of 100 nm and a subsequent sapphire (Al_2_O_3_) layer with a thickness of 16 nm were deposited on a silicon wafer (Si(100)) covered with a native oxide layer via sputtering and atomic layer deposition. Next, rectangular Au blocks with a transverse length of 30 μm, a thickness of 100 nm, and a longitudinal length (*L*) ranging from 50 nm to 220 nm were placed on the Al_2_O_3_/Au film using standard electron beam lithography and thermal evaporation methods. The entire area of the sample was coated with dye (coumarin 343)-doped poly(methyl methacrylate) (PMMA) using a standard spin-coating technique. The thickness of the PMMA layer was determined using a spectroscopic ellipsometer. To prevent unintentional loss of fluorescence intensity due to the bleaching of dye molecules, the dye-doped PMMA layer was occasionally recoated throughout the experiment. The thickness of the PMMA layers was 50 nm for the experiments shown in Figures 2 and 4 and Supplementary Movie 1, and 60 nm for Figure 3. The uncertainty of the thickness was 5 nm, including the nonuniformity of the film. The difference of 5 nm in the film thickness resulted in a difference of approximately 0.1 × 10^8^ m/s in the group velocity of the SPPs under the experimental conditions used in the study.

## Supplementary Material

Supplementary Material Details
