# Supplementary material for: Femtosecond imaging of spatial deformation of surface plasmon polariton wave packet during resonant interaction with nanocavity
Source: Nanophotonics. 2022 Feb 25;11(7):1321–33. doi: 10.1515/nanoph-2021-0740 (PMC11501654; doi:10.1515/nanoph-2021-0740)
Supplement: Supplementary file 5 — Supplementary Material Details [file j_nanoph-2021-0740_suppl.pdf]

Supporting information - Femtosecond imaging of spatial deformation of surface plasmon polariton wave packet during resonant interaction with nanocavity

Naoki Ichiji[1], Yuka Otake[1], Atsushi Kubo[2,*]

[1]Graduate School of Pure and Applied Sciences, University of Tsukuba, 1-1-1 Tennodai, Tsukuba-shi, Ibaraki 305-8571, Japan
[2]Faculty of Pure and Applied Sciences, University of Tsukuba, 1-1-1 Tennodai, Tsukuba-shi, Ibaraki 305-8571, Japan

Section S1. Transmittance spectra

The transmittance spectra of metal–insulator–metal nanocavities (MIM-NCs) are shown in Fig. 1(c) of the main text. These spectra were obtained by taking the Fourier transforms of the temporal waveforms of surface electric fields calculated by a finite-difference time-domain (FDTD) method, as described below.

The commercial software Poynting for Optics (Fujitsu) was used. Figure S1 shows the schematic of the simulation models. Figures S1(a) and S1(b) show the cavity and reference models, respectively. In the cavity model, a Au block was placed on the surface of an Al_2O_3 layer to form a cavity structure at a distance $d = 4$ µm from an excitation wave source. The cavity length L was sequentially varied from 10 nm to 400 nm. In addition, the entire surface was covered with a poly(methyl methacrylate) (PMMA) layer with a thickness of 60 nm to simulate the experimental conditions. The relative dielectric constants of Au and Al_2O_3 were determined using the Drude–Lorentz model [1] and the nondispersive constant ($\epsilon_{\mathrm{Al_2O_3}} = 2.75$), respectively.

The function of the waveform shown in Fig. S1(c) is expressed as

$$f = \exp\left(-\left(\frac{t - t_0}{\Delta\tau}\right)^2\right)\sin(\Omega t), \quad \text{(S1)}$$

where $\Delta\tau$ is 1.2 fs, t_0 is 30 fs, and Ω is π rad/fs.

Two observation points (a, b) were chosen in each model to record the temporal evolution of the out-of-plane electric field ($E_z(t)$). For the cavity models (Fig. S1(a)), a was set at the right edge of the cavity to record transient cavity resonances, and b was set at a position 7 µm away from the excitation wave source (p) to record transmission waves. The reference waves for each cavity model were prepared by setting the coordinates of an (a and b) pair in the reference model to be identical to those in the corresponding cavity model (Fig. S1(b)).

The spatial distributions of the out-of-plane electric field (E_Z) of a surface plasmon polariton (SPP) wave packet (WP) interacting with an MIM-NC (L = 160 nm) were simulated using the FDTD method (Fig. S1(d)). MIM-NCs support eigenmodes in which the wavelengths are considerably shortened according to the dispersion relation of the antisymmetric waveguiding mode of an MIM multilayer. When L coincides with multiples of half the eigenmode, the field is enhanced owing to the Fabry–Pérot resonance.

This system can be regarded as an optical cavity coupled with a waveguide, and the incident SPP WP couples to the cavity mode and is subsequently released from the other side when the frequency component coincides with the eigenenergy of the cavity. In this study, the resonant spectrum of an MIM-NC, $R(\omega)$, was evaluated as

$$R(\omega) = \frac{\left|F_{Cav_a}(\omega)\right|^2}{\left|F_{Ref_a}(\omega)\right|^2}, \quad \text{(S2)}$$

where $F_{Cav_a}(\omega)$ and $F_{Ref_a}(\omega)$ are the fast Fourier transforms of $E_z(t)$ measured at a in the cavity and reference models, respectively. In a similar manner, the transmittance spectrum of an MIM-NC, $T(\omega)$, was defined as shown in Fig. 1(c) of the main text by using $E_z(t)$ measured at b in the respective models:

$$T(\omega) = \frac{\left|F_{Cav_b}(\omega)\right|^2}{\left|F_{Refb}(\omega)\right|^2}. \quad \text{(S3)}$$

The resonance spectra, $R(\omega)$, were plotted as functions of L and the energy (Fig. S1(e)). For the MIM-NC with L = 160 nm, the energy of the carrier wave of the excitation source (1.52 eV) agreed well with the energy of the second-order cavity mode. The Poynting vector flowed from the flat area of the Au surface into the cavity; thus, a considerable part of the SPP WP was squeezed into the insulator layer of the MIM-NC (inset of Fig. S1(d)).

The transmitted SPP WP obtained at b contained not only the component that passed through the insulator layer of the cavity but also the component that propagated along the upper surface of the Au block of the MIM-NC, where continuous energy bands were supported. Consequently, the interference of the two paths caused the lineshape of the transmittance spectrum of an MIM-NC to have a Fano-type asymmetric shape. The resonance spectra (Fig. S1(e)) exhibited symmetric lineshapes and peak energies (open circles) essentially determined by the dispersion relation of the SPP of the MIM waveguiding mode and the resonance order. However, the transmittance spectra (Fig. 1(c) of the main text) showed that the first-order resonance (second- and third-order resonances) was more enhanced for the lower (higher) energy side. Notably, the transmittance spectrum was considerably depressed in the area between the first- and second-order resonances. At the energy ranges corresponding to the femtosecond laser used in this study, the transmittance decreased to approximately 0.1 for a cavity of length $L \approx 100$ nm, suggesting that the MIM-NC functions as a subwavelength SPP reflector. Conversely, color-selective transmissions are expected for longer L values.

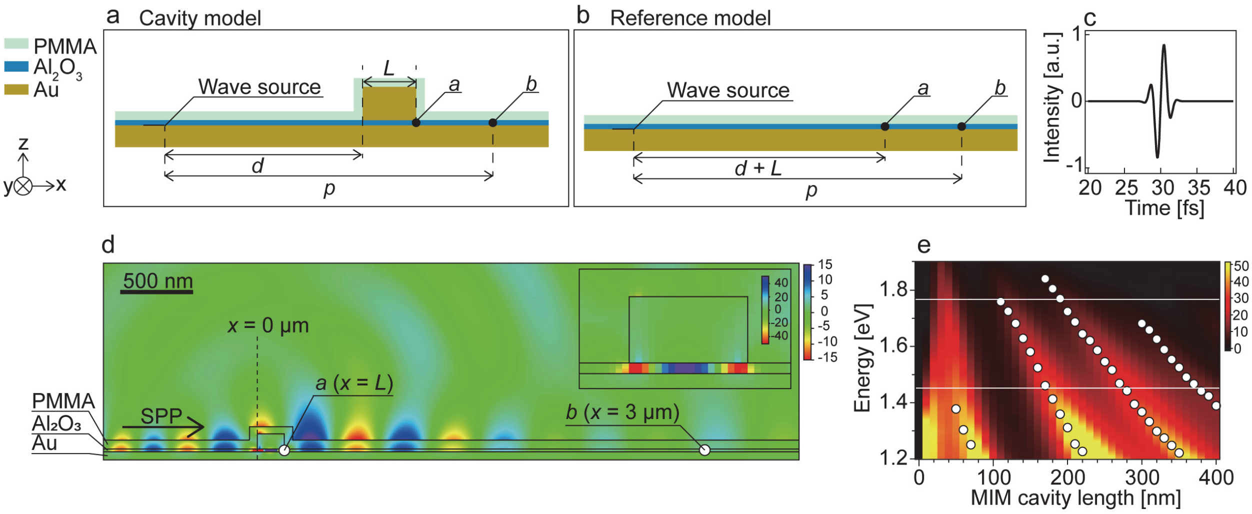

Fig. S1. Schematic of multilayered structure models used for FDTD simulations. (a) Cavity model. (b) Reference model. (c) Waveform of excitation wave. (d) Electric field (E_z) distribution at second-order resonance of MIM nanocavity with length of 160 nm calculated by FDTD simulation. (e) Two-dimensional plots of resonance spectral intensity of MIM nanocavity shown as function of length of cavity and energy. The four sets of solid circles show the peaks for the first- to fourth-order resonance modes of the MIM nanocavities, and the horizontal white lines indicate the full-width-at-half-maximum lines of the laser used in the experiment.

Section S2. Complex dispersion (CD) model

This section provides the details of the CD model. Figure S2(a) shows the calculation of an SPP WP excited by a laser pulse. The evolution of the SPP WP was calculated by evaluating the amplitudes and phases of the Fourier components of the SPP WP for $x = 0$– 150 μm. An SPP WP excited at $x = 0$ was expanded to a polar Fourier series as $E_{SPP}(0,t) = \sum_i R(0,\omega_i)\exp\big(i(\omega_i t + \phi(0,\omega_i))\big)$, where $R(0,\omega_i)$ and $\phi(0,\omega_i)$ are the amplitude and initial phase, respectively, of a Fourier component with an angular frequency ω_i. In this study, the excitation source was assumed to be a transform-limited pulse having a spectrum identical to that of the femtosecond laser used in the experiment. The SPP WP was expected to duplicate the waveform of the excitation pulse, except for a phase retardation between light and the SPP [2,3].In this model, we divide the model region into three regions: the before-cavity, in-cavity, and after-cavity regions. The length of the before cavity region, d, and that of the entire area, u, were 40 and 150 μm, respectively.

First, $E_{SPP}(x,t)$ of the SPP WP propagating on the flat plane was expressed using the polar Fourier expansion coefficients evaluated for x as

$$E_{SPP}(x,t) = \sum_i R(x,\omega_i)\exp\big(i(\omega_i t + \phi(x,\omega_i))\big). \tag{S4}$$

In the before-cavity region $(x < d)$, The amplitude $R(x,\omega_i)$ and phase $\phi(x,\omega_i)$ of the Fourier component $R(x,\omega_i)\exp\big(i(\omega_i t + \phi(x,\omega_i))\big)$ were calculated using the CD relation (complex wave vector) of the SPP mode as follows [2,3]:

$$\phi(x,\omega_i) = \phi(0,\omega_i) + k'_{SPP}(\omega_i)x, \tag{S5}$$

$$R(x,\omega_i) = R(0,\omega_i) \times \exp(-k''_{SPP}(\omega_i)x), \tag{S6}$$

where k'_{SPP} and k''_{SPP} are the real and imaginary parts, respectively, of the complex SPP wave vector $k_{SPP}(\omega_i) = k'_{SPP}(\omega_i) + ik''_{SPP}(\omega_i)$. The waveform of the SPP WP was retrieved by performing the inverse Fourier transform of the obtained Fourier components (Fig. S2(a)). The derivation of the complex SPP wave vector in the system considered in this study is described in Section S5.

Next, we discuss the effects of the interaction between the SPP WP and MIM NC in the in-cavity region ($d \leq x \leq d + L$). To incorporate the resonance interaction, the following assumptions were applied to the model. First, the dominant channel used by the SPP to pass through the in-cavity region was assumed to be the MIM waveguide mode accompanied by resonances. Another path for overcoming the in-cavity region through the top surface of the MIM-NC was considered in the spectral shape of the transmission. Second, the propagation through an MIM-NC was assumed to be phenomenologically modeled by Lorentz resonators with multiple eigenfrequencies and an additive phase shift that represented the phase accumulation through the waveguiding mode.

The model was formulated as follows. The waveform of the Lorentz-oscillator response, E_{Lor}, was expressed as follows using the electric field of the SPP at $x = d$, $E_{SPP}(t)|_{x=d}$:

$$E_{Lor}(t) = \sum_{i=1}^{5} \int_{-\infty}^{\infty} E_{SPP}(t)|_{x=d} \cdot \mathrm{e}^{-\gamma_i(t-t')} \sin[f_i(t - t')]\, dt', \tag{S7}$$

where γ_i is the inverse of the coherence time of the Lorentz resonator (all γ_i values were set to 1/5 fs^{-1}) and f_i is the angular frequency of the i-th-order resonance [4]. The γ_i values and f_i value for each resonance mode were determined from the FDTD simulation. In this model, five oscillators were set to include all the resonance modes that could be relevant to the transmission in the energy range of 1.2–1.9 eV and the cavity length L = 10–400 nm (Fig. 1(c) of the main text). The SPP WP in the MIM waveguide at $x = d$, $E_{cav}(t)|_{x=d}$, was defined as $E_{cav}(t)|_{x=d} = A \cdot E_{Lor}(t)$, where A is a constant that aligns the amplitudes of $E_{SPP}(t)|_{x=d}$ and $E_{cav}(t)|_{x=d}$.

The phase accumulation for each Fourier component attributable to the in-cavity region ($d < x < d + L$) was evaluated by simply replacing the term of the SPP wave vector in Eq. (S5) with the term of the wave vector of an MIM (Fig. S5).

To retrieve the waveform of an SPP WP at the exit of the MIM-NC ($x = d + L$), the amplitudes of the Fourier components, $R(d + L, \omega_i)$, were calculated by multiplying the amplitudes at $x = d$ by the transmittance spectrum $T(\omega)$, which was evaluated by the FDTD method in Section S1. Thus, the spectral filtering effect of an MIM-NC was considered [5].

Further phase accumulations and amplitude attenuations in the after-cavity region ($d + L < x < u$) are again evaluated by the term of SPP wave vector as before-cavity region. Therefore, for the range $(d + L) < x$, the phase $\phi(x, \omega_i)$ and amplitude $R(x, \omega_i)$, including the all contributions of propagation and cavity-SPP resonances, were expressed as

$$\phi(x, \omega_i) = \phi_0 + \phi_{Disp}(x) + \phi_{Lor}, \tag{S8}$$

$$R(x, \omega_i) = R_0 \times \eta_{\mathrm{Disp}}(x) \times T(\omega). \tag{S9}$$

where $\phi_{Disp} = k'_{SPP}(\omega_i)(x - L) + k'_{MIM}(\omega_i)L$ and $\eta_{Disp} = \exp(-k''_{SPP}(\omega_i)(x - L)) \times \exp(-k''_{MIM}(\omega_i)L)$ are the phase advancements and the intensity attenuation caused by the propagation through the area constructed by both the flat area and MIM-NC, respectively.

The waveform of the SPP WP, $E_{SPP}(x, t)$ ($d + L < x < u$), was obtained by performing the inverse Fourier transform.

The propagation calculation shown in Fig. S2 (a) can be applied not only to the electric field of the SPP WP, E_{SPP}, but also to the electric field of the light WP propagating in a dispersive medium by replacing the SPP wave vector k_{SPP} with the wave vector obtained from the refractive index of the medium. In Fig. 5 of the main text, the chirped excitation pulse was obtained by performing the calculation shown in Fig. S2(a) using the dielectric constant of fused silica glass [6]. Moreover, it will be possible to determine complex SPP mode indices in devices, which are composed by composite materials or specific waveguiding structures, by setting the complex SPP wave vector as the unknown variable in the CD model and comparing the calculation results with the time-resolved fluorescent microscopy, as shown in Fig. 4(a,b) in the main text.

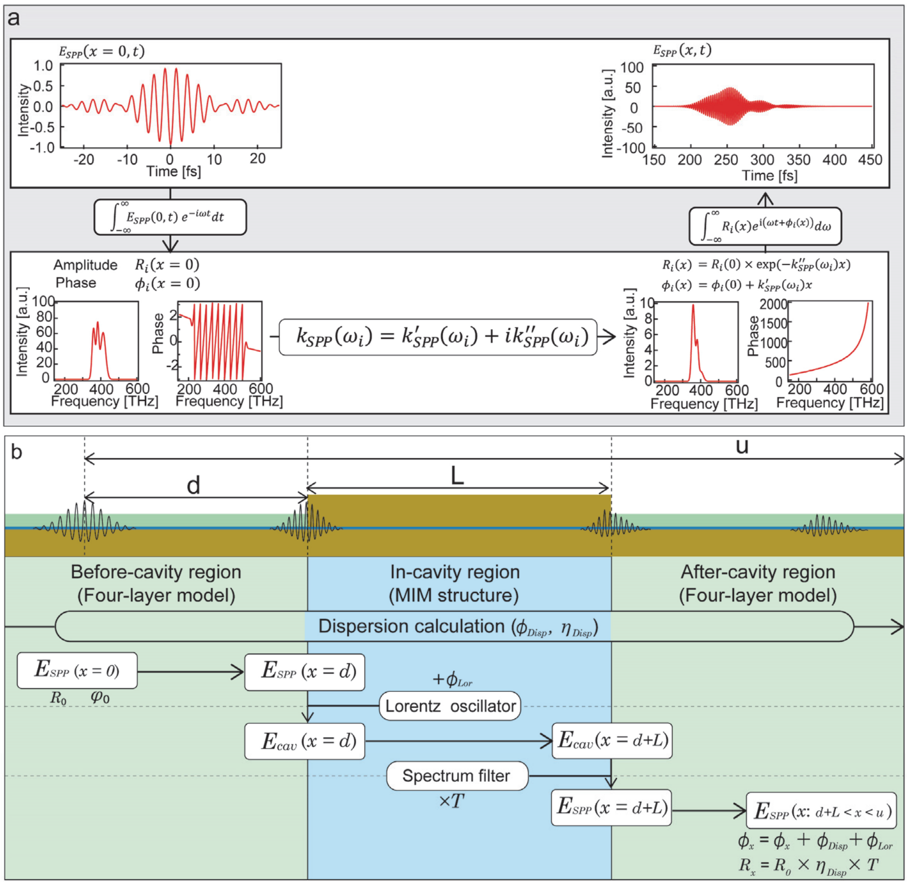

Fig. S2. (a) Calculations for propagation of SPP. (b) Entire calculation process of CD model.

Section S3. Intensity distribution obtained by two-photon fluorescence microscopy

Section S2 describes the acquisition of the temporal waveform of the transmitted SPP WP. However, the temporal waveform was not directly observed by fluorescence microscopy. In this section, the calculation for the conversion of a temporal waveform to a spatial fluorescence intensity waveform is described. The observed images shown in Fig. 2 represent the intensity of fluorescence from the dye molecules covering the sample surface. In this calculation, the fluorescence intensity from a dye molecule was defined to be proportional to the fourth power of the electric field [7,8].

Assuming that the exposure period of the CCD camera was sufficiently long, the fluorescence intensity $I_{Exp}(x)$ at an arbitrary position in the image was obtained as

$$I_{Exp}(x) = \int_{-\infty}^{\infty} \left(E_{Total}(x,t)\right)^4 dt, \tag{S10}$$

where E_{Total} is the total electric field on the sample surface and comprises contributions from the pump light, probe light, and SPP WP.

To obtain the corresponding result, the following calculation was performed:

$$I_{Calc}(x) = \int_{-\infty}^{\infty} \left(|E_{SP}(x,t) + E_{Light}(x,t)|^2\right)^2 dt, \tag{S11}$$

where E_{SP} and E_{Light} are the electric fields of the SPP WP and laser pulse in the CD model, respectively. Typical calculation results are shown in Fig. S3. Figure S3(a) shows the space–time trajectories for E_{SPP} and E_{Light}. Two intersections of the probe light with the SPP WP excited by the pump light were confirmed. The interference at these intersections is reflected as beat patterns in the fluorescence intensity graph (Fig. S3(b)). The calculated fluorescence intensity graph shown reveals distinct beat patterns that possess the WPs-like envelope at positions corresponding to the intersections in Fig. S3(a). The beat patterns in the forward and backward regions show different wavelengths and widths because of the opposite propagation direction of the SPP WP.

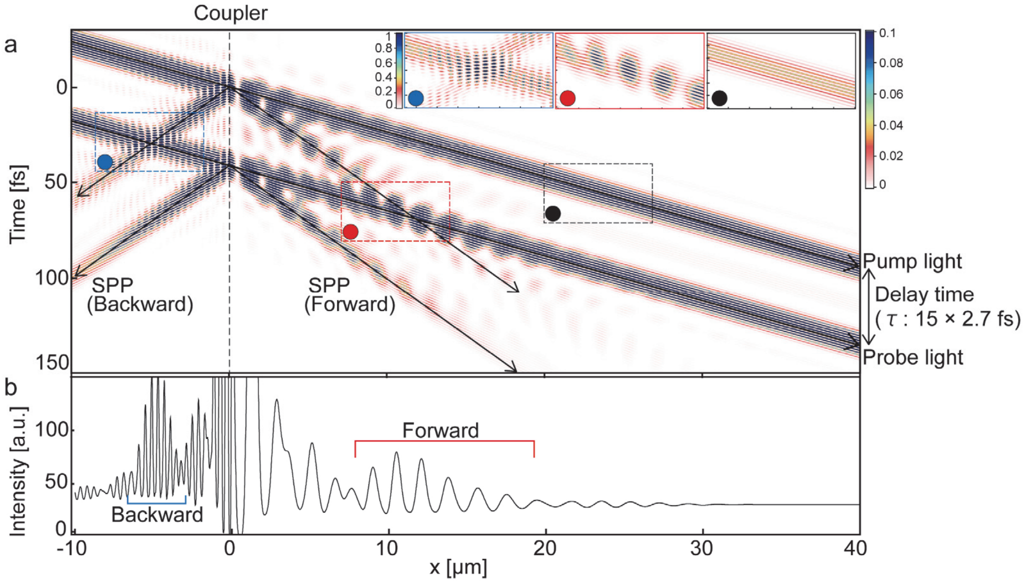

Fig. S3. Intensity distribution obtained by two-photon fluorescence microscopy. (a) Calculated trajectories for E_{SPP} and E_{Light} (pump light and probe light) for pump–probe delay corresponding to phase $15.0 \times 2\pi$ of carrier frequency. The vertical dashed line indicates the position of a light–SPP coupler. Inserts show the expanded image of the feature area. Different color scales were used to increase the visibility of the fringes. (blue) Interference between backward propagation SPP WP and probe light, (red) Interference between forward propagation SPP WP and probe light, and (black) pump light itself without interference. (b) Calculated fluorescence intensity obtained from (a).

Section S4. Removal of background and delay time independent components

In this study, a two-photon fluorescence microscopy method was used. This method can observe the interference beat between a pump-excited SPP WP and a probe pulse. However, the obtained images contain background information in addition to the target beat pattern, for example, the fluorescence intensity distribution caused by the nonuniformity of the dye concentration and laser intensity. In particular, the interference beat between the pump-excited SPP and the pump pulse itself has a considerably greater intensity than the beat of the SPP WP–probe interference. Therefore, a differential image was generated from two images acquired with different delay times to remove the background information.

Figures S4(a) and S4(b) show the reference area of the fluorescence images obtained at delay times $\tau_{d1} = 31.25 \times 2.7$ fs and $\tau_{d2} = 31.25 \times 2.7$ fs. The time 2.7 fs corresponds to a cycle of the carrier wave of the laser. Whereas the pump–pump interference beat at $x = 0$ μm and the overall intensity distribution agree in the two graphs, the beat patterns at $x = 20$ μm show an antiphase relationship. The cross sections of the two images shown in Fig. S4(c) clearly show the antiphase relationship. Figure S4(d) shows an extracted fluorescence image obtained by taking the difference between the two images in Figs. S4(a) and S4(b) (subtracting τ_{d1} from τ_{d2}, $\overline{\tau_d} = 31.5 \times 2.7$ fs). The cross section of the extracted image is shown in Fig. S4(e). At $x = 20$ μm, the extracted beat pattern preserves the spatial waveform constructed by the SPP WP–probe interference beat. As shown in Fig. 4 of the main text, the same operation was performed on the results of the CD model to extract the beat patterns for comparison with the experimental results.

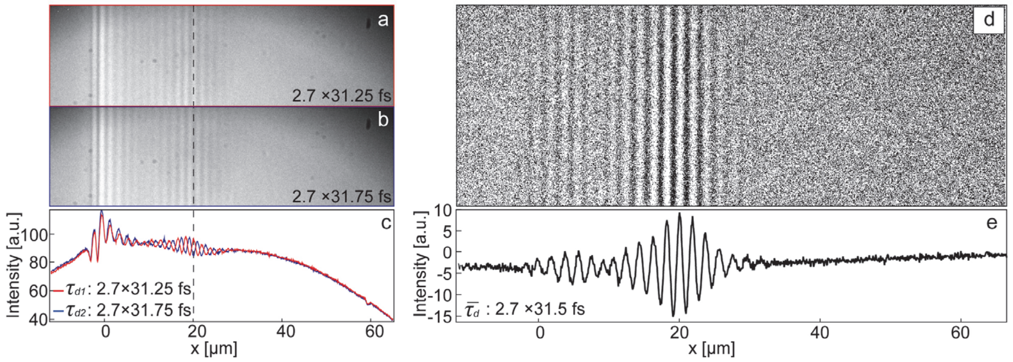

Fig. S4. Removal of background. (a, b) Fluorescence microscopy images obtained at delay times of (a) 31.25 × 2.7 fs (red) and (b) 31.75 × 2.7 fs (blue). (c) Cross sections of beat patterns in fluorescence images in (a) (red) and (b) (blue). (d) Interference beat between propagating SPP WP and probe light extracted by taking difference between two graphs in (a) and (b). (e) Cross section of beat pattern in difference image shown in (d).

Section S5. CD curve

This section describes the derivation of the CD curves used in the CD model. The CD model requires two dispersion curves: one for the plane area without a cavity and the other for the cavity area with an MIM waveguide structure.

First, to evaluate the plane-area dispersion curve, the SPP wave vector (k_{spp}) was calculated using the four-layer model proposed by Pockrand [9]. Pockrand described a second-order theoretical approximation of the SPP dispersion relation for coated metal surfaces. This approximation corresponds well to the experimental and simulation results when the coating layer is sufficiently thin (≤ 100 nm). This model assumes that the sample consists of four layers: a Si substrate, a Au film (100 nm), an insulator layer consisting of an Al_2O_3 film and a PMMA layer (76 nm in total), and air.

The dispersion relation of the cavity area was derived using the MIM structure model as follows [10]. The focus was on the so-called odd mode, where the spatial distribution of E_x (E_z) is antisymmetric (symmetric) with respect to the center ($z = 0$) of the MIM structure. The length of the structure was assumed to be infinite. The wave functions of the electromagnetic field that propagated in the x direction from the wave vector k and frequency ω were expressed as follows:

For $|z| > H/2$ (in the metal layer),

$$H_y^m(\boldsymbol{r},\omega) = A\frac{\omega\epsilon_0\epsilon_m(\omega)}{k}\exp(-k_m|z|)\exp[i(kx-\omega t)]\,, \tag{S12}$$

$$E_x^m(\boldsymbol{r},\omega) = -\frac{ik_m}{k}\frac{|z|}{z}A\exp(-k_m|z|)\exp[i(kx-\omega t)]\,, \tag{S13}$$

$$E_z^m(\boldsymbol{r},\omega) = A\exp(-k_m|z|)\exp[i(kx-\omega t)]\,, \tag{S14}$$

and for $|z| < H/2$ (in the insulator layer),

$$H_y^i(\boldsymbol{r},\omega) = 2B\frac{\omega\epsilon_0\epsilon_i(\omega)}{k}\cosh(k_iz)\exp[i(kx-\omega t)]\,, \tag{S15}$$

$$E_x^i(\boldsymbol{r},\omega) = i2B\frac{k_i}{k}\sinh(k_iz)\exp[i(kx-\omega t)]\,, \tag{S16}$$

$$E_z^i(\boldsymbol{r},\omega) = 2B\cosh(k_iz)\exp[i(kx-\omega t)]\,, \tag{S17}$$

where ϵ_0 is the dielectric constant of vacuum; ϵ_m (ω) and ϵ_i are the relative dielectric constants of the metal (Au) and insulator (Al_2O_3), respectively; A and B are arbitrary constants; the subscripts and superscripts m and i indicate the metal and insulator, respectively; and k_m and k_i are the z components of the wave vector, respectively, and are written as $k_m = \sqrt{k^2 - \frac{\omega^2}{c^2}\epsilon_m(\omega)}$ and $k_i = \sqrt{k^2 - \frac{\omega^2}{c^2}\epsilon_i(\omega)}$. The application of boundary conditions to connect Eqs. (S12) and (S15) using Eqs. (S14) and (S16) at $z = H/2$ resulted in

$$\epsilon_m(\omega)A\exp(-k_m|y|) = 2B\epsilon_i\cosh(k_iy) \tag{S18}$$

and

$$-k_mA\exp(-k_m|y|) = 2Bk_i\sinh(k_iy)\,. \tag{S19}$$

The eigenequation of the MIM structure was derived from Eqs. (S18) and (S19) as follows:

$$-\frac{k_m}{k_i} = \frac{\epsilon_m(\omega)}{\epsilon_i}\tanh\left(k_i\frac{H}{2}\right) \tag{S20}$$

The relationship between the wave vector k or the wavelength $\lambda_{MIM} = 2\pi/k$ and the frequency ω, i.e., the dispersion relation of the SPP in the MIM structure, was obtained by numerically solving Eq. (S20).

Figure S5(a) shows the real part of the calculated dispersion curves. The dashed curve was obtained using the four-layer model, whereas the solid curves were obtained using the MIM structure models with different insulator layer thicknesses. The curves obtained using the MIM structure model exhibited a greater inclination than the curve obtained using the four-layer model. In addition, the dispersion curve obtained using the MIM structure model moved toward larger wavenumbers as the thickness decreased. Similarly, the imaginary parts of the dispersion curves were plotted, as shown in Fig. S5(b). The dispersion curves obtained using the four-layer model and MIM structure model increased in value as the frequency increased.

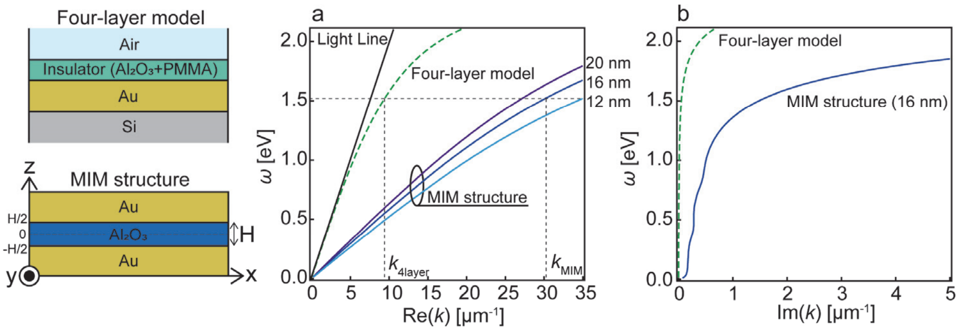

Fig. S5. (a) Real and (b) imaginary parts of dispersion curves of SPP for insulator-layer-covered Au calculated using four-layer model (dashed line) and MIM structure model with Al_2O_3 thicknesses H = 12, 16, and 20 nm (solid lines).

Section S6. Finite integration technique (FIT) simulation

An FIT simulation was performed under the conditions corresponding to those used for the CD model to confirm the validity of the calculation results obtained using the CD model. In this simulation, the commercial software CST Studio Suite (Dassault Systèmes) was used. Figure S6(a) shows a schematic of the entire area of the FIT simulation. A periodic boundary condition was applied to the y axis, and absorbing boundary conditions were applied to the x and z axes. An MIM nanocavity was formed by placing a Au block with a height of 100 nm on an Al_2O_3 film. The cavity length L ranged from 0 to 380 nm. A model with $L = 0$ nm, i.e., a model without a cavity, was defined as the reference model. As in the experimental model, a ridge structure (light–SPP coupler) was placed 40 μm away from the cavity. Both the length and height of the ridge structure were 100 nm. The entire surface was covered with a PMMA layer with a thickness of 60 nm. The relative dielectric function of Au determined by Rakic et al. [1] was used, with one Drude term and four Lorentz terms in the Drude–Lorentz model. The relative dielectric constants of PMMA ($\epsilon_{PMMA} = 2.34$) and Al_2O_3 ($\epsilon_{Al_2O_3} = 2.75$) were set as nondispersive constants. The excitation pulse was a Fourier-limited Gaussian pulse with a center wavelength of 800 nm and a time duration of 10 fs. The angle of incidence was 45° from the surface normal.

Figure S6(b) shows the spatial waveform of the propagated SPP WP obtained from the E_z component of the reference model. The up-chirping of the carrier frequency occurred owing to the propagation on the metal surface. The spectrogram shows the up-chirping more clearly. Figure S6(c) shows the space–frequency spectrogram of the E_z field shown in Fig. S6(b). The stretched contour distribution from the upper left to the lower right reflects the up-chirping of the SPP WP. The spectrogram profile of the reference model shown in Fig. S6(c) shows an overall continuous shape with no specific peaks or dips.

In contrast to the spectrograms acquired using the reference model, those acquired using the cavity models showed characteristic distributions depending on the cavity length. Figures S6(d)–S6(i) show the space–frequency spectrograms of the SPP WPs for $L = 60–160$ nm at intervals of 20 nm. The sequence of the spectrograms in Figs. S6(d)–S6(f) shows that the peak position shifted from the center to the lower right as the cavity length increased. This shift can be explained by the redshift of the first-order resonance of the cavity. The peak frequency of a transmission component shifts with the eigenfrequency of a cavity because an MIM-NC acts as a Fabry–Pérot-type spectrum filter that passes spectral components falling within the resonance linewidth. Figure S6(f) shows a new peak in the upper left in addition to the existing peak in the lower right. The new peak is the transmission component attributable to second-order resonance. Across the sequence shown in Figs. S6(f)–S6(i), the new peak gradually moves towards the lower right direction as the intensity increased, whereas the initial peak disappeared.

It should be noted that the calculation conditions for the CD model are different from those shown in Fig. 4 in the main text. While the CD model in the main text uses the Fourier-limited pulse of the experimentally obtained laser spectrum as the excitation source, the CD model in this section uses a 10 fs Gaussian-shaped pulse, which is the same as the excitation wave source in the FIT simulation. The amplitude and peak position of an SPP WP were examined without considering the interference with a probe pulse used to calculate the beat patterns. As shown in Figs. S7(a) and S7(b), the results from the FIT simulation and CD model showed good agreement. These results confirm the validity of the CD model.

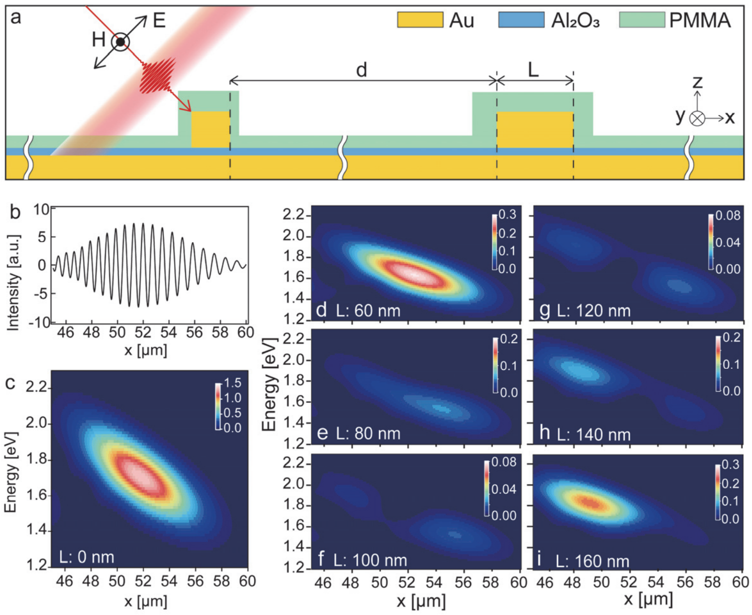

Fig. S6. FIT simulation results. (a) Schematic of model used for FIT simulations. (b) Spatial waveform of SPP WP obtained from E_z component of reference model. A snapshot was taken when the SPP WP propagated by approximately 50 μm. (c) Space–frequency spectrogram of (b). The color scale represents the intensity. (d)–(i) Space–frequency spectrograms of SPP WPs obtained for simulation models containing cavity with L = 60–160 nm.

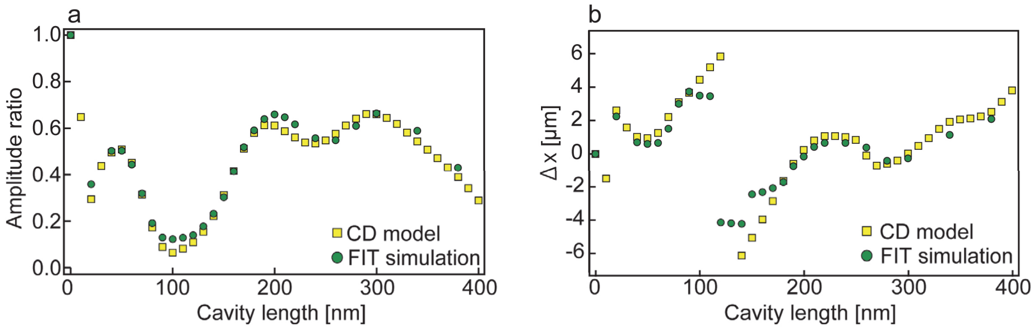

Fig. S7. Comparison of FIT simulation and CD model results. (a) Ratio of peak amplitude of transmitted SPP WP to that of reference WP. (b) Peak shift of transmitted SPP WP compared with that of reference WP. The yellow squares and green circles represent results obtained using the CD model and FIT simulation, respectively.

References

[1] A. D. Rakić, A. B. Djurisic, J. M. Elazar, M. L. Majewski, "Optical properties of metallic films for vertical-cavity optoelectronic devices," Appl. Opt., vol. 37, pp. 5271–5283, 1998.

[2] A. Kubo, N. Pontius, H. Petek, "Femtosecond microscopy of surface plasmon polariton wave packet evolution at the silver/vacuum interface," Nano Lett., vol. 7, pp. 470-475, 2007.

[3] L. Zhang A. Kubo, L. Wang, H. Petek, T. Seideman, "Imaging of surface plasmon polariton fields excited at a nanometer-scale slit," Phys. Rev. B, vol. 84, pp. 245442, 2011.

[4] B. Lamprecht, J. R. Krenn, A. Leitner, F. R. Aussenegg, "Resonant and off-resonant light- driven plasmons in metal nanoparticles studied by femtosecond-resolution third-harmonic generation," Phys. Rev. Lett., vol. 83, pp. 4421–4424, 1999.

[5] X. Zhao, J. Zhang, K. Fan, G. Duan, J. Schalch G. R. Keiser, R. D. Averitt, X. Zhang, "Real-time tunable phase response and group delay in broadside coupled split-ring resonators," Phys. Rev. B, vol. 99, pp. 245111, 2019.

[6] I. H. Malitson, "Interspecimen comparison of the refractive index of fused silica," J. Opt. Soc. Am., vol. 55, pp. 1205-1209, 1965.

[7] T. Hattori, A. Kubo, K. Oguri, H. Nakano, H. T. Miyazaki, "Femtosecond laser-excited two-photon fluorescence microscopy of surface plasmon polariton," Jpn. J. Appl. Phys., vol. 51, pp. 04DG03, 2012.

[8] Hattori, T.; Kubo, A.; Oguri, K.; Nakano, H.; Miyazaki, H. T. Imaging of surface plasmon polaritons using femtosecond laser excited two-photon fluorescence microscopy," Rev. Laser Eng., vol. 40, pp. 603, 2012.

[9] I. Pockrand, "Surface plasma oscillations at silver surface with thin transparent and absorbing coatings," Surf. Sci. vol. 72, pp. 577-588, 1978.

[10] N. Ichiji, Y. Otake, and A. Kubo, "Spectral and temporal modulations of femtosecond SPP wave packets induced by resonant transmission/reflection interactions with metal-insulator-metal nanocavities," Opt. Express, vol. 27, pp. 22582-22601, 2019.
